# Nucleotide binding domain and leucine-rich repeat pyrin domain-containing protein 12: characterization of its binding to hematopoietic cell kinase

**DOI:** 10.7150/ijbs.41798

**Published:** 2020-03-05

**Authors:** Yue Zhang, Curtis T. Okamoto

**Affiliations:** Department of Pharmacology and Pharmaceutical Sciences, School of Pharmacy, University of Southern California, USA 90089-9121

**Keywords:** yeast two-hybrid, innate immunity, protein‐protein interaction, NLRP12, HCK, acute myeloid leukemia

## Abstract

Protein-protein interactions are key to define the function of nucleotide binding domain (NBD) and leucine-rich repeat (LRR) family, pyrin domain (PYD)-containing protein 12 (NLRP12). cDNA encoding the human PYD + NBD of NLRP12 was used as bait in a yeast two-hybrid screen with a human leukocyte cDNA library as prey. Hematopoiesis cell kinase (HCK), a member of the c-SRC family of non-receptor tyrosine kinases, was among the top hits. The C-terminal 40 amino acids of HCK selectively bound to NLRP12's PYD + NBD, but not to that of NLRP3 and NLRP8. Amino acids F503, I506, Q507, L510, and D511 of HCK are critical for the binding of HCK's C-terminal 40 amino acids to NLRP12's PYD + NBD. Additionally, the C-terminal 30 amino acids of HCK are sufficient to bind to NLRP12's PYD + NBD, but not to its PYD alone nor to its NBD alone. In cell lines that express HCK endogenously, it was co- immunoprecipitated with stably expressed exogenous NLRP12. Also, NLRP12 co-immunoprecipitated and co-localized with HCK when both were overexpressed in 293T cells. In addition, in this overexpression system, steady-state NLRP12 protein expression levels significantly decreased when HCK was co-expressed. Bioinformatic analysis showed that *HCK* mRNA co-occurred with *NLRP12* mRNA, but not with other *NLRP* mRNAs, in blood and marrow samples from acute myeloid leukemia (AML) patients. The mRNA of *NLRP12* is also co-expressed with *HCK* in AML patient samples, and the levels of mRNA expression of each are correlated. Together these data suggest that NLRP12, through its binding to HCK, may have an effect on the pathogenesis of AML.

## Introduction

Pattern recognition receptors (PRR) are the first major line of cellular defense against damage- associated molecular patterns (DAMP) and pathogen- associated molecular patterns (PAMP) 1. Among PRR, one class of proteins is the nucleotide-binding domain (NBD) and leucine-rich repeat (LRR) receptor (NLR) family. NBD and LRR PYD - containing protein 12 (NLRP12) is one protein that belongs to the PYD subfamily of the NLR class and is a multi-functional protein 2. NLRP12's functions likely all depend on defined protein-protein interactions. For example, in anti-inflammatory functions of NLRP12, NLRP12 interacts with tumor necrosis factor receptor associated factor 3 (TRAF3) and nuclear factor kappa-light-chain-enhancer of activated B cells (NF-κB)-inducing kinase (NIK) in the non-canonical NF-κB pathway, when toll-like receptors (TLR) are stimulated with Pam3Cys4 triacylated lipopeptides followed by addition of cluster of differentiation 40 (CD40) 3. NLRP12's inhibition of the non-canonical NF-κB pathway, thereby effecting an anti- inflammatory function, has been reported in many publications 7-11. For example, in colitis-driven colon cancer, *Nlrp12* knock-out (KO) mice experience a higher frequency of death, weigh less, have a shorter colon length, and have more severe disease progression than wild type (WT) mice, all of which may occur through NLRP12's inhibition of the non- canonical NF-κB pathway 3,4. In addition, *Nlrp12* KO mice have been reported to exhibit higher innate immunity when it is infected by the virus because NLRP12 inhibits the non-canonical NF-κB pathway; thus, in KO mice, a more significant increase of NF- κB activity is observed compared to WT mice when it is infected by the virus 5. And in the case of NLRP12 protecting the diversity of intestinal microbiota 6, thereby preventing obesity 7, it has also been reported that *Nlrp12* KO mice display significantly decreased intestinal microbial diversity due to increased innate immunity, resulting from the lack of inhibition of the non-canonical NF-κB pathway.

In addition, NLRP12 also interacts with interleukin (IL)-1 receptor-associated kinase 1 (IRAK1) in the canonical NF-κB pathway when TLRs are stimulated, also to inhibit the inflammatory response 8. Kanneganti's group also showed that NLRP12 expression downregulated the canonical NF-kB pathway during *Salmonella* infection 4,9. In contrast, for human studies of NLRP12-associated autoinflammatory disorders, no protein-protein interactions have been reported 10,11. Functionally, however, *Nlrp12* KO mice had a higher immunological response in the autoimmune model of contact hypersensitivity and defective migration of their dendritic cells (DC) 12; thus, these mice are more prone to develop contact hypersensitivity. In addition, Luken et al. demonstrated that NLRP12 impacts the experimental autoimmune encephalomyelitis (EAE) model via regulating T-cell responses 13. But, in these mouse studies, no protein-protein interactions had been reported either 12. And NLRP12 is protective against *Yersinia pestis* infection through NLRP12's activation of the inflammasome which results in secretion of IL-18 and IL-1β. But, no protein-protein interactions of NLRP12 within inflammasomes had been characterized 14. Also, NLRP12 also regulates the c-Jun N-terminal kinase (c-JNK) pathway in hepatocytes 15; but, again, no protein-protein interactions within the NLRP12-JNK pathway had been demonstrated. Thus, there is a further need to find and to characterize previously unreported NLRP12-binding proteins. Knowing NLRP12's protein binding partners may help to gain mechanistic insight into pathways previously characterized as being regulated by NLRP12. Moreover, identifying and validating potentially novel binding partners of NLRP12 may suggest novel functions for NLRP12.

In humans, NLRP12 is mainly expressed in macrophages, DCs, and neutrophils. In mice, NLRP12 is primarily expressed in DCs and neutrophils 5,12,16. The NLRP12 protein has three domains, from N- to C-terminus: PYD, NBD, and LRR domains 2,17,18. It is generally accepted that the LRR domain, like NLRP1's LRR domain and NLRP3's LRR domain, is responsible for sensing ligands or other types of activators of NLRPs in modulating their role in an immunologic response 19-22. However, interestingly, it has been reported that without the LRR domain, NLRP3 still can be activated by triggers of the canonical NF-κB pathway 23. The NBD domain is responsible for self-oligomerization when NLRP is activated, and the PYD domain is responsible for regulating downstream signaling pathways (and thus, it is called the “interaction domain”) 16,23. Here we used a truncated form of NLRP12 (PYD + NBD) to identify possible protein-protein interactions that may regulate signaling pathways in which NLRP12 is involved. In addition, to our knowledge, there are currently no protein-protein interaction studies that have been performed for this truncated form of NLRP12 and may thus represent a new general approach to screening for protein-protein interactions involved in signaling complexes with NLRPs.

A yeast two-hybrid screen was performed to identify potential binding partners of NLRP12's PYD + NBD. HCK, a member of the c-SRC family of non-receptor tyrosine kinases, was one of the top positive clones (“hits”) among all of the proteins that appeared to interact with NLRP12. Further experiments were done to confirm the novel binding of NLRP12 to HCK and to characterize key structural features of the binding interaction. In addition, co-expression of HCK with NLRP12 resulted in a significant decrease of steady-state NLRP12 protein expression levels. Finally, together with a bioinformatics analysis included here, a potential interaction between NLRP12 and HCK may be involved in modulating the role of HCK in the progession of AML 24-26.

## Results

### NLRP12 interacts with HCK in the yeast two-hybrid assay

**Figure 1:**
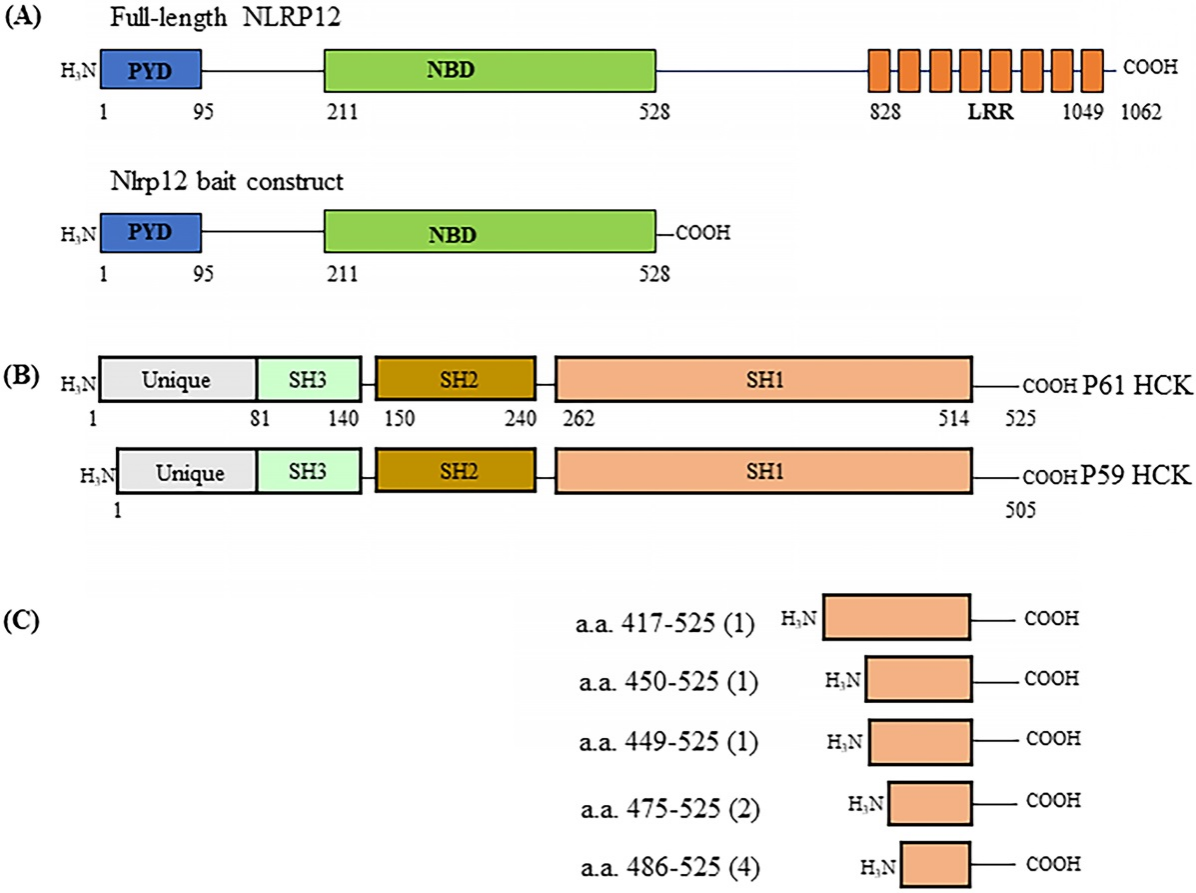
(A) Schematic drawing of the domain structure of full-length NLRP12 (top) and the domains used as the construct for the yeast two-hybrid screen (bottom). PYD: Pyrin domain; NBD: Nucleotide binding domain; and LRR: Leucine rich repeat domain. (B) The domain structures of the two alternatively spliced isoforms of HCK, HCK p59 and HCK p61. (C) Identification of the clones of *HCK* interacting with *NLRP12* (“hits”) obtained from the yeast two-hybrid screen. Among the nine of the clones positive for HCK, one clone encoded for the smallest interacting fragment of HCK, the C-terminal 40 amino acids of HCK. The numbers in parentheses indicate the total number of hits obtained in the screen.

A human leukocyte cDNA library was used as prey in the screening of binding partners for NLRP12 using the yeast two-hybrid assay. The basic principle of the yeast two-hybrid assay is that if the cDNA library contains clones that express proteins, or parts thereof, that interact with the co-expressed bait, the NLRP12 PYD + NBD (Figure 1A) in this case, then the co-transformed yeast clones will grow. The cDNA clones from the library that supported growth would then be isolated and characterize, to identify what proteins interact with NLRP12's PYD + NBD. The leukocyte library was used because NLRP12 is mainly expressed in leukocytes in humans 2,16,27, and thus putative binding partners for NLRP12 are also likely to be expressed there. The screening resulted in 119 positive clones from the leukocyte cDNA library (“hits”) expressed from 48 distinct genes Supplementary materials, Table S1 that were subsequently identified as encoding for proteins (or portions thereof) that potentially interact with NLRP12's PYD + NBD. The clones with the most hits are listed in Table 1. Among the 119 hits, which included repeated hits of the same genes, 9 were individual clones that encoded fragments of a member of the c-SRC family of non-receptor tyrosine kinases, HCK, which was among the highest number of hits for a single gene (Figure 1B and Table 1). Moreover, of the 9 positive *HCK* clones, 5 encoded distinct HCK fragments (Figure 1C). Full-length *HCK* p61 was also obtained as a hit in another independent yeast two-hybrid screen (data not shown). HCK has two isoforms, p59 and P61, in Homo sapiens. P61 and p59 share the same transcript, but p61 uses an uncommon start codon CTG, and p59 uses the common one, ATG 28, to give rise to two distinct transcripts. Exogenously expressed in phagocytes, HCK p59 is mainly localized to the plasma membrane, while HCK p61 is primarily localized to lysosomes, and both of them are also found in the Golgi apparatus 29. It has been suggested that overexpression of p59 promotes the formation of membrane protrusions such as pseudopodia and overexpression of p61 induces podosome rosettes and triggers actin polymerization around lysosomes 29,30. However, there are no differences between p61 and p59 in the region of HCK that appears to bind to NLRP12's PYD + NBD (see below), the C-terminal part of HCK. Thus, detecting a full-length clone of only p61 as a hit, opposed to p59, appeared to be coincidental.

### In directed screens using the yeast two-hybrid assay, NLRP12's PYD + NBD domain binding to HCK C-terminal 40 amino acids appears to be selective for NLRP12, and HCK binding to NLRP12 appears to be selective for HCK

**Figure 2:**
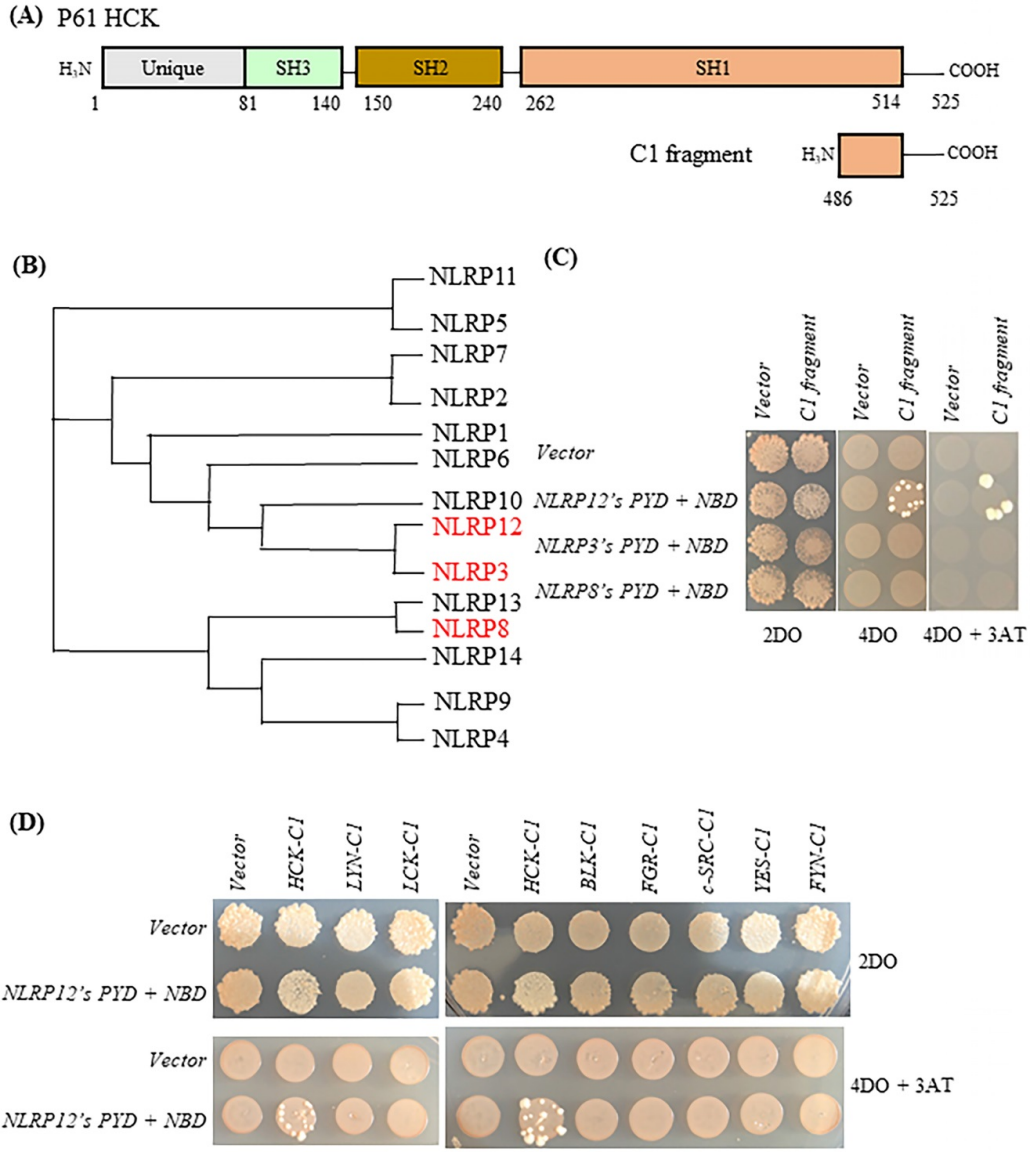
Selectivity of binding of NLRP12 with HCK. (A) Schematic drawing of HCK (p61 isoform, top) and, for comparison, the region of HCK (“HCK C1 fragment,” lower) used for testing the selectivity of binding of HCK to other members of the NLRP family of proteins. The numbers represent the amino acid residues of the protein or protein fragment. (B) Phylogenetic tree showing the relationships among the PYD + NBD region of all of the members of the human NLRP family of proteins. The phylogenetic tree was produced through Clustal Omega (https://www.ebi.ac.uk/Tools/msa/clustalo/). For each NLRP family member, the PYD + NBD of the longest isoform was selected for generating the phylogenetic tree. NLRP3 is phylogenetically the closest NLRP to NLRP12, while NLRP8 is phylogenetically among the most distant from NLRP12. NLRP3 and NLRP8 are highlighted in red color. (C) When *NLRP12's PYD + NBD* and *HCK C1 fragment* were co-transformed into yeast, they grew on the highest stringency plates (4DO + 3AT), consistent with an interaction between the two constructs. On the other hand, neither *NLRP3's PYD + NBD* co-transformed with the C1 fragment nor *NLRP8's PYD + NBD* co-transformed the C1 fragment grew on the highest stringency plates. These results are consistent with a selective interaction between NLRP12 and the HCK C1 fragment, but not with the other NLRPs. (D) Yeast clones grew on the high stringency plates 4DO + 3AT when *HCK's C1 fragment* and *NLRP12's PYD + NBD* were co-transformed, but they did not grow on the high stringency 4DO + 3AT plates when the C1 fragments of other members of the c-SRC family of non-receptor tyrosine kinases were co-transformed with *NLRP12's PYD + NBD*. These results are again consistent with a selective interaction between NLRP12 and HCK.

Initially, the HCK C-terminal 40 amino acids (“HCK C1 fragment”) was the shortest HCK fragment from a positive clone that interacted with the bait, *NLRP12's PYD + NBD* (Figure 2A). NLRP 3 and NLRP8 were chosen based on the phylogenetic trees of the PYD + NBD domains of members of the NLRP family: phylogenetically, NLRP3's PYD + NBD is the most closely related NLRP PYD + NBD to that of NLRP12, while NLRP8's PYD + NBD is among the furthest related PYD + NBD to that of NLRP12 (Figure 2B). In Figure 2C, yeast clones grew on the 4 drop out (DO) (synthetic dropout (SD) plus amino acids supplements minus tryptophan, leucine, histidine, and adenine (-Trp-Leu-His-Ade)) and 4DO + 3-Amino-1,2,4-triazole (3-AT) high stringency plates when the yeast were co-transformed with *NLRP12's PYD + NBD* and *HCK C1 fragment*; and thus, this result is indicative of an interaction between the two constructs. However, yeast clones were not observed on 4DO and 4DO + 3-AT plates when *NLRP3's PYD + NBD* and *HCK C1 fragment* were co-transformed, nor when *NLRP8's PYD + NBD* and *HCK C1 fragment* were co-transformed. These results suggest that the HCK C1 fragment binds to NLRP12's PYD + NBD, but not to those from either NLRP3 or NLRP8, results consistent with a selective interaction between NLRP12's PYD + NBD and the HCK C1 fragment.

Similarly, yeast clones again grew on the high stringency 4DO and 4DO + 3-AT plates when the *HCK C1 fragment* was co-transformed with *NLRP12's PYD + NBD*. However, clones did not grow on the 4DO and 4DO + 3-AT plates when the *C1 fragment of other members of the c-SRC family of non-receptor tyrosine kinases* were co-transformed with *NLRP12's PYD + NBD* (Figure 2D). This experiment suggests that the interaction of HCK C1 fragment with NLRP12 is selective for HCK; the C1 fragment of other members of the c-SRC family of non-receptor tyrosine kinases did not bind to NLRP12's PYD + NBD (Figure 2D).

### In directed screens using the yeast two-hybrid assay, amino acids F503, I506, Q507, L510, and D511 of HCK are critical for the binding between NLRP12's PYD + NBD and HCK C-terminal 40 amino acids

**Figure 3:**
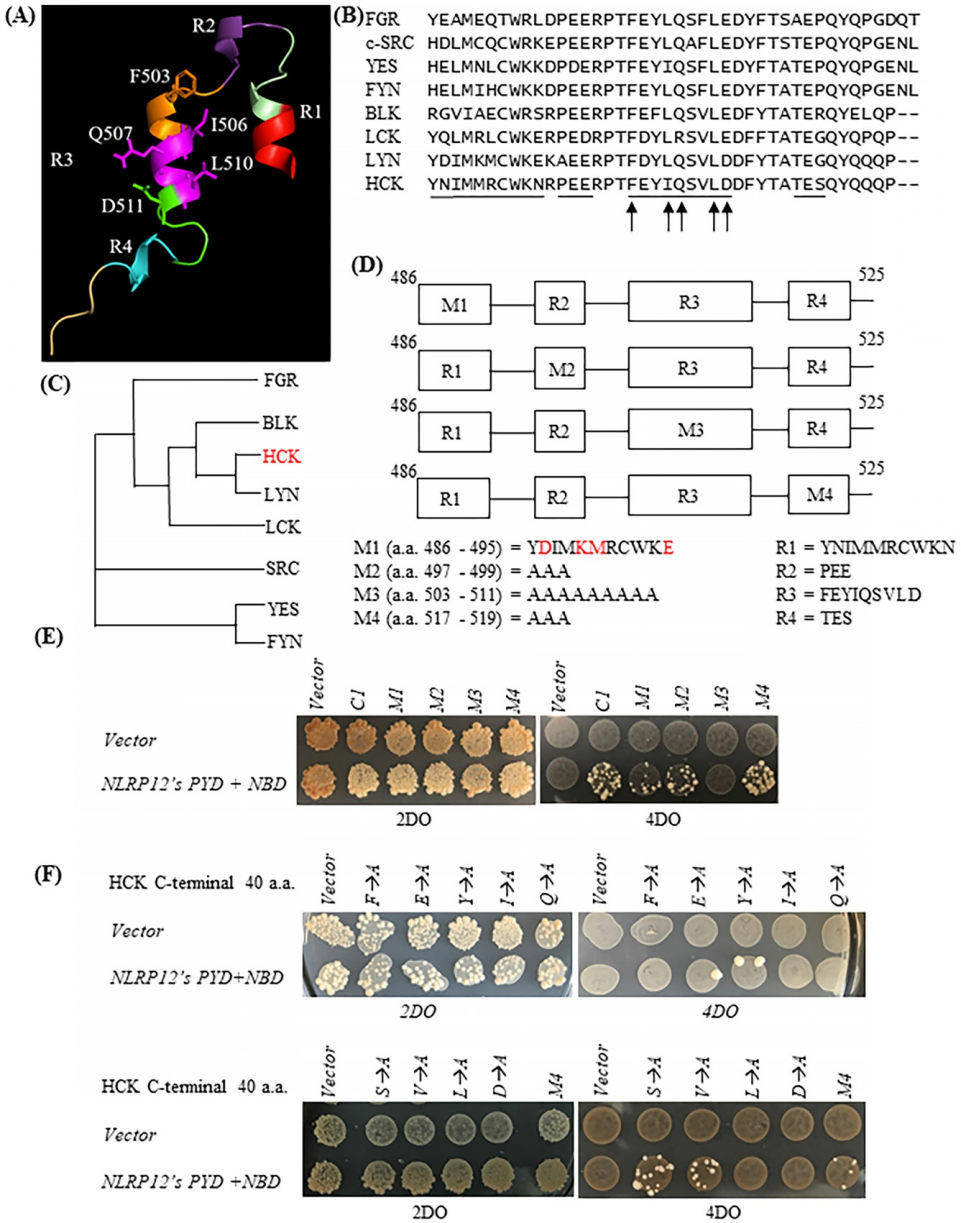
(A) Structure of the HCK C1 fragment generated by Chimera from the University of California, San Francisco (version 1.13.1), extracted from its structure within the entire HCK protein. HCK C1 fragment was thus divided into four helical domains: R1, R2, R3, and R4. The side chains of amino acids F503, I506, Q507, L510, and D511 are shown. However, it is not clear whether the C1 fragment retains the structural features shown here when it expressed on its own. (B) Primary amino acid sequence alignments showing of the C1 fragments of all members of the c-SRC non-receptor tyrosine kinase family (40 amino acids for HCK, BLK, LCK, and LYN; 42 amino acids for FGR, c-SRC, YES, and FYN). The black lines indicate the R1, R2, R3, or R4 regions. The arrows denote the five critical amino acids for binding of HCK C1 fragment to NLRP12's PYD + NBD. (C) Phylogenetic tree showing the relationship among all of the C1 fragments of the members of the c-SRC family of non-receptor tyrosine kinases. The phylogenetic tree was produced through Clustal Omega (https://www.ebi.ac.uk/Tools/msa/ clustalo/). HCK is highlighted in red color. (D) Four sets of distinct mutations were made within the HCK C1 fragment. M1: the amino acids in the R1 region were mutated to the amino acids identical to the R1 region in LYN (mutated amino acids are highlighted in red color); M2: the amino acids in the R2 region were all mutated to alanine (Ala); M3: the amino acids in the R3 region were all mutated to Ala; and M4: the amino acids in the R4 region were all mutated to Ala. (E) Yeast did not grow on high stringency 4DO + 3AT plates when *HCK C1 fragment with the M3 mutations* was co-transformed with *NLRP12's PYD + NBD*. But yeast can grow on the high stringency 4DO + 3AT plates when the *HCK C1 fragment contained either the M1, M2, or M4 mutations*, suggesting that the R3 region within the C1 fragment was necessary for binding to NLRP12 NBD + PYD. (E) Yeast did not grow on high stringency 4DO plates when *NLRP12's PYD + NBD* was co-transformed with *HCK C1 fragment with the nucleotides' product* that has single Ala substitutions at either F503, I506, Q507, L510, or D511. But yeast grew on high stringency 4DO plates when *NLRP12's PYD + NBD* was co-transformed with *HCK's C1 fragment with the nucleotides' product* in which the following amino acids in the R3 region were individually mutated to Ala: E504, Y505, S508, or V509.

The HCK C1 fragment was divided into four helical regions according to its atomic structure 31 (protein data bank (PDB) accession number: 1AD5): R1, R2, R3, and R4 (designated by us) (Figure 3A). As shown in the sequence alignments in Figure 3B, the primary amino acid sequences of R2, R3, and R4 are very well conserved among members of the c-SRC family of non-receptor tyrosine kinases, while R1 is not. To characterize further the important structural features of HCK's binding to NLRP12's PYD + NBD, all of the amino acids comprising each of the R2, R3, and R4 domains were mutated to alanine, but only one section at a time was mutated, and these were denoted as the HCK M2 fragment, HCK M3 fragment, and HCK M4 fragment, respectively. The amino acids comprising the R1 region were mutated to those amino acids in the R1 region of the c-SRC family non-receptor tyrosine kinase LYN, which has the closest phylogenetic relationship to HCK (Figure 3C). All of the mutated constructs are illustrated in Figure 3D. Yeast still grew on the high stringency 4DO + 3AT plates when *NLRP12's PYD + NBD* was co-transformed with the *HCK M1 fragment, HCK M2 fragment,* and* HCK M4 fragment*, respectively. But yeast could not grow when *NLRP12's PYD + NBD* was co-transformed with *HCK M3 fragment* (Figure 3E). These experiments suggested that the R1, R2, and R4 regions are dispensable for binding, while R3 may be important for binding. Further analysis was conducted to identify individual critical amino acids of HCK's R3 region binding to NLRP12's PYD + NBD using alanine scanning mutagenesis. However, the intervening sequences between R1, R2, R3, and R4 were not examined for binding.

To identify the key amino acid residues involved in binding of R3 to NLRP12's PYD + NBD, each amino acid in R3 was individually mutated to alanine in an alanine scanning mutagenesis analysis, on the background of the HCK C1 fragment. As shown by the yeast two-hybrid assay, the mutations of *F503A*,* I506A*,* Q507A, L510A*, and* D511A* abolished the growth of yeast clones on the high stringency 4DO when these yeast strains were co-transformed with *NLRP12's PYD + NBD* (Figure 3F). These experiments suggest that these five amino acids are critical for binding. On the other hand, yeast clones still grew on the high stringency 4DO plates when the *HCK C1 fragments* with the *E504A*, *Y505A*, *S508A*, or *V509A* mutations were co-transformed with *NLRP12's PYD + NBD* (Figure 3F). These experiments suggest that E504A, Y505, S508, and V509 are dispensable for binding of HCK C1 fragment to NLRP12's PYD + NBD. Overall, however, these results suggest that the amino acids F503, I506, Q507, L510, and D511 in HCK are critical for the binding of HCK C1 fragment to NLRP12's PYD + NBD.

On the other hand, YES also has these five amino acid residues (Figure 3B), suggesting that, while they are important for binding, they cannot account for the specificity or selectivity of binding between NLRP12 and HCK. In addition, the amino acids R500, P501, T502, D512, T515, Q520, and Y521, are 100% conserved among all other members of the c-SRC family of non-receptor tyrosine kinases and are therefore unlikely to play a role in specificity and selectivity of binding of NLRP12 with HCK. But, it is possible that two or more amino acids in the intervening sections between the R1-R4 regions, which were not analyzed by mutagenesis, may then actually account for the specificity and selectivity of binding of HCK to NLRP12. One example is that the combination of R496, P497, and Q522 is unique to HCK compared to other members of the c-SRC family of non-receptor tyrosine kinases.

### In directed screens using the yeast two-hybrid assay, the shortest fragment of HCK that binds to NLRP12's PYD + NBD is its C-terminal 30 amino acids

**Figure 4:**
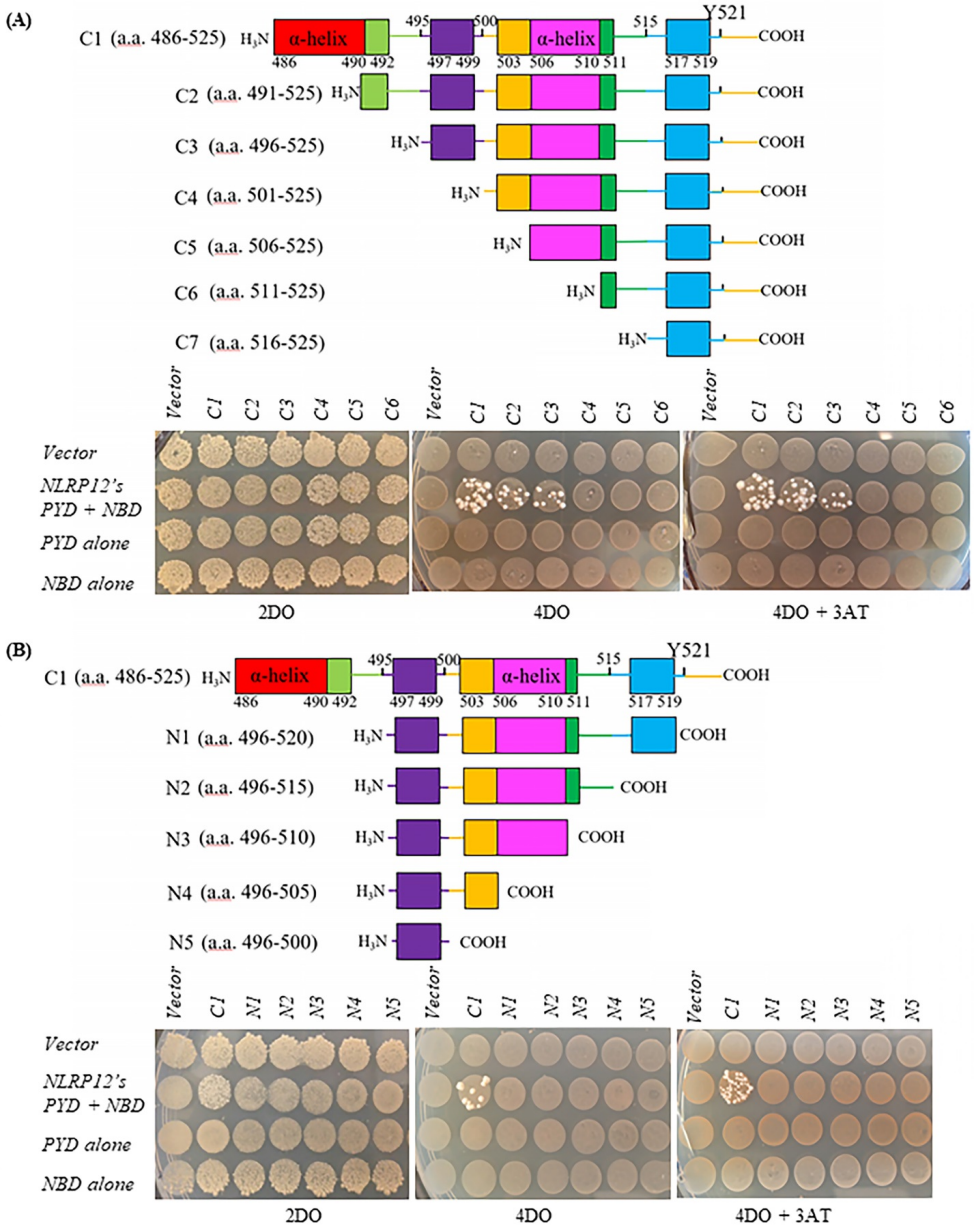
(A) Top: schematic drawing of HCK truncations comprising the C1 to C7 fragments. Bottom: yeast grew on the high stringency 4DO plates and 4DO +3AT plates when *HCK's C-terminal 40 amino acid fragment (C1)*, *HCK's C-terminal 35 amino acid fragment (C2)*, and *HCK's C-terminal 30 amino acid fragment (C3)*, individually, were co-transformed with *NLRP12's PYD + NBD*. But yeast did not grow on the high stringency 4DO plates nor 4DO+ 3AT plates when *C4 to C7 fragments* were co-transformed to with *NLRP12's PYD + NBD*. The lack of yeast growth upon co-transformation of the *C7 fragment* and *NLRP12* are not shown. (B) Top: schematic drawing of Hck truncations comprising the N1 to N5 fragments. Bottom: yeast grew on the high stringency 4DO plates and 4DO +3AT plates when *HCK's C-terminal 40 amino acid fragment (C1)* was co-transformed with *NLRP12's PYD + NBD (control)*. But yeast did not grow on the high stringency 4DO plates nor 4DO+ 3AT plates when *N1 and N5 fragments* were co-transformed with *NLRP12's PYD + NBD*, suggesting that the very C-terminal 5 amino acids are also necessary for binding of NLRP12. The colored boxes represent different fragments that were deleted each time.

The shortest form of HCK that bound to NLRP12's PYD + NBD was determined by observing whether the yeast clones grew on high stringency 4DO or 4DO +3AT plates when *NLRP12's PYD + NBD* was co-transformed with *HCK fragments C1* to *C7* and *N1* to *N5*, respectively (Figure 4) (data of *NLRP12's PYD + NBD* co-transformed with *HCK fragment C7* not shown). HCK's C1 to C7 fragments were gradually truncated from the N-terminus of the HCK C-terminal 40 amino acid fragment (Figure 4A). HCK's N1 to N5 fragments were gradually truncated from the C-terminus of the HCK C-terminal 40 amino acid fragment (Figure 4B). Yeast strains grew on high stringency 4DO or 4DO +3AT plates when either the *HCK C1, C2,* or* C3* fragment was co-transformed with *NLRP12's PYD + NBD*. Thus, of all of the constructs screened, the shortest fragment of HCK that can apparently bind to NLRP12's PYD + NBD is the C-terminal 30 amino acids, as indicated by the HCK C3 fragment (Figure 4). In addition, the last C-terminal 5 amino acids of the HCK C-terminus also appear to be important for binding, as clones co-transformed with *NLRP12's PYD + NBD* with *N1*, with a deletion of the C-terminal 5 amino acids of *HCK*, did not grow on the high stringency 4DO and 4DO + 3AT plates (Figure 4B). The structures of both inactive HCK (PDB accession number: 1AD5) and active c-SRC (PDB accession number: 1Y57), the prototypical member of this family of nonreceptor tyrosine kinases related to HCK, show that the last C-terminal 4 amino acids of c-SRC are surface-exposed, indicating that these four amino acids that are critical for binding are available to interact with NLRP12 32,33.

### NLRP12 co-immunoprecipitates with HCK from mammalian cells, and NLRP12 is co-localized with HCK in mammalian cells

**Figure 5:**
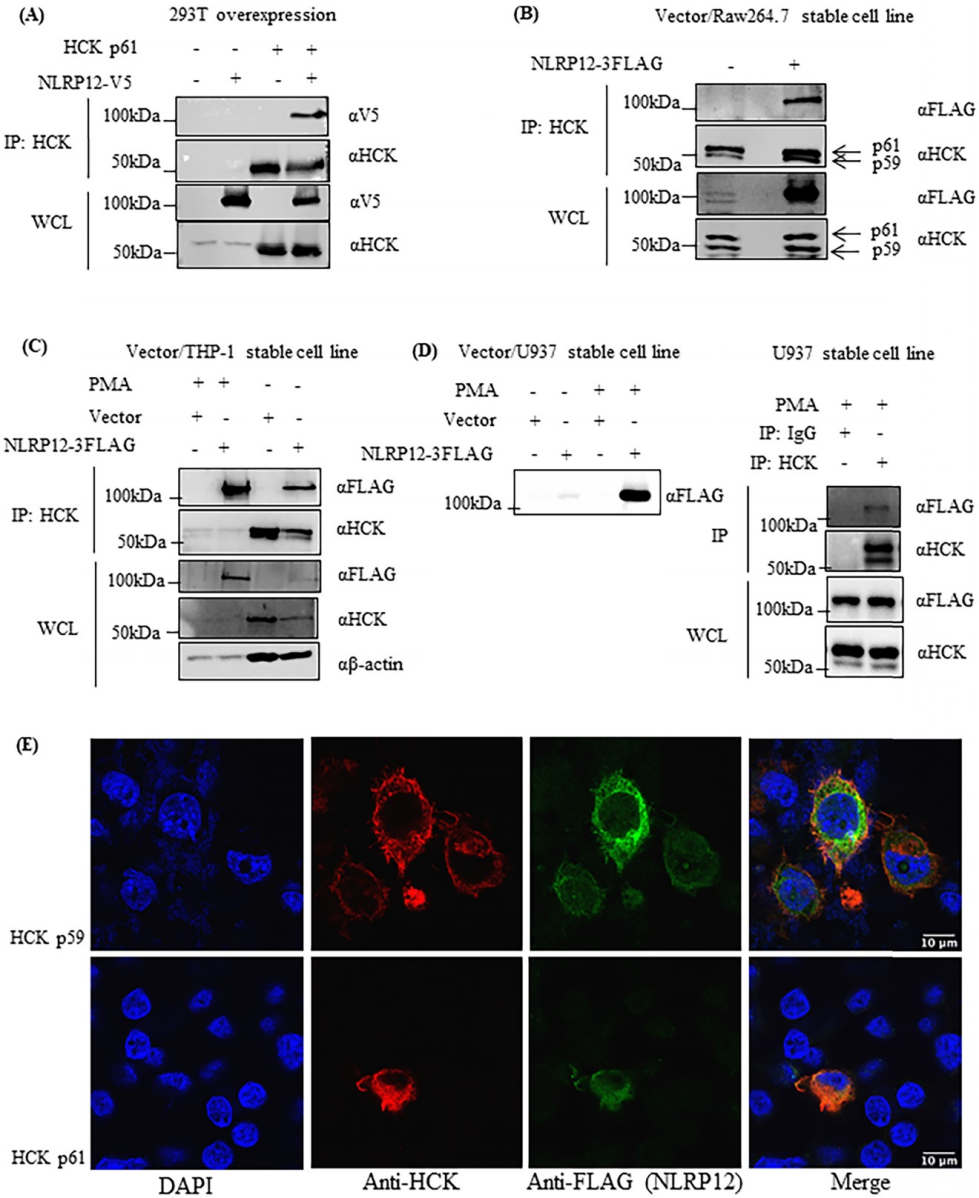
NLRP12 co-immunoprecipitates with HCK and co-localizes with HCK by immunofluorescence. (A) NLRP12 co-immunoprecipitated with HCK when both proteins were transiently exogenously co-expressed in 293T cells, with NLRP12 having to be expressed as epitope-tagged forms. Cells were lysed using a non-denaturing cell lysis buffer (see Material and Methods). Co-immunoprecipitations were done using a mouse monoclonal anti-HCK antibody followed by protein A/G agarose. The immunoprecipitated proteins were analyzed by immunoblotting. A mouse monoclonal anti-V5 antibody was used for detecting NLRP12-V5, while a mouse monoclonal anti-HCK antibody was used for detecting HCK. The same method was used for experiments shown in (B), (C), and (D). (B) NLRP12 co-immunoprecipitated with endogenous HCK in macrophage-like RAW 264.7 cells, when NLRP12 is exogenously and stably expressed. Arrows show the two isoforms of HCK p59 and p61 that were immunoprecipitated by the anti-HCK antibody, although it is not clear whether NLRP12 co-immunopreciptitated with one form or the other, or both. A mouse monoclonal anti-FLAG antibody was used for detecting NLRP12-FLAG, while a mouse monoclonal anti-HCK antibody was used for detecting HCK. (C) Exogenously and stably expressed NLRP12 co-immunoprecipitated with endogenously expressed HCK in monocyte-like THP-1 cells. In these cells, phorbol 12-myristate 13-acetate (PMA) (1µM) was added to enhance the expression of NLRP12 under the PMA-sensitive cytomegalovirus promoter of the *NLRP12* plasmid. A mouse monoclonal anti-FLAG antibody was used for detecting NLRP12-FLAG; a mouse monoclonal anti-HCK antibody was used for detecting HCK; and a mouse monoclonal anti- β actin was used as the protein loading control. (D) Left panel shows that NLRP12 was exogenously and stably expressed in lymphoblast-like U937 cells. Right panel shows that NLRP12 co-immunoprecipitated with endogenously expressed HCK in U937 cells after PMA was added to 1µM, as was done for THP-1 cells, to enhance expression of NLRP12. In addition, a mouse monoclonal anti-IgG antibody was used for immunoprecipitation as a negative control. A mouse monoclonal anti-FLAG antibody was used for detecting NLRP12-FLAG, while a mouse monoclonal anti-HCK antibody was used for detecting HCK. (E) Immunofluorescent images showing the colocalization of a cohort of NLRP12 with either HCK p59 or p61. HCK was immunolabeled with a mouse monoclonal anti-HCK antibody, followed by a secondary goat anti-mouse IgG (H+L) cross-adsorbed, conjugated to Alexa Fluor 488. For NLRP12, the primary antibody was a rabbit monoclonal anti-FLAG antibody, and the secondary antibody was a goat anti-rabbit IgG (H+L) cross-adsorbed secondary antibody, conjugated to Alexa Fluor 555**.** Nuclei were counterstained by DAPI.

NLRP12 and HCK could be co-immunoprecipitated when they were both heterologously co-expressed in 293T cells (Figure 5A). Also, semi-endogenous conditions were used for co-immunoprecipitations from cells in which HCK is endogenously expressed, and NLRP12 was heterologously stably expressed in epitope-tagged form, such as in macrophage-like RAW 264.7 cells (Figure 5B), monocyte-like THP-1 cells (Figure 5C), and lymphocyte -like U937 cells (Figure 5D). Semi-endogenous conditions have been used in previous reports where NLRP12 was shown to interact with TRAF3 3, NIK 34, and IRAK1^8^. In all cases, NLRP12 co-immunoprecipitated with the p59 and p61 isoforms of HCK, but from these experiments, it is not clear that whether NLRP12 interacts with p59, p61, or both isoforms of HCK. In U937 cells and THP-1 cells, since heterologous NLRP12 expression level was low, adding phorbol 12-myristate 13-acetate (PMA) (1 µM for 24 hr) increased NLRP12 expression level by stimulating the PMA-sensitive cytomegalovirus promoter of *NLRP12*'s expression plasmid (Figure 5C and 5D). Taken together, these data suggest that NLRP12 and HCK (either p59, p61, or both isoforms) can interact with each other in a mammalian cell system, a result that in part validates the findings from the yeast two-hybrid assay.

After heterologous co-expression of NLRP12 with either the p61 or the p59 isoform of HCK in 293T cells, immunofluorescent staining of NLRP12 and HCK showed that NLRP12 can be co-localized with either p61 or p59 isoforms of HCK in most of the 293T cells (Figure 5E and Supplementary materials, Figure S1). The co-localization of NLRP12 and HCK is consistent with an intracellular interaction between the two proteins. In addition, the lack of selectivity of NLRP12 co-localizing with either p61 or p59 isoform of HCK is consistent with the results from the screening assay, where the putative binding region of HCK to NLRP12 is identical in both the p61 and p59 isoforms.

### The steady-state level of NLRP12 protein may decrease when HCK protein is co-expressed

**Figure 6:**
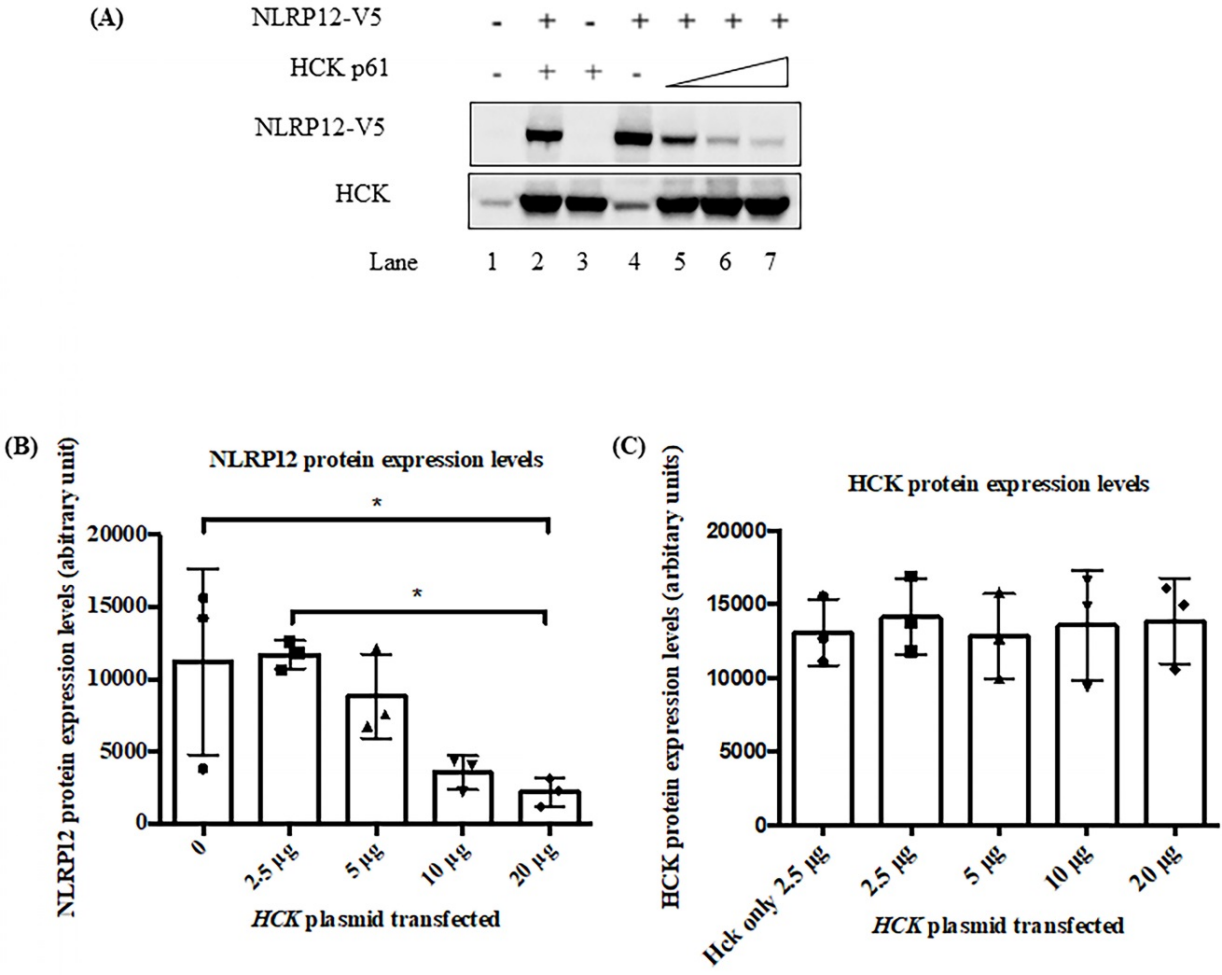
The co-expression of HCK protein significantly decreased the steady-state protein expression levels of NLRP12. (A) Epitope-tagged NLRP12-V5 and HCK p61 were co-transfected into 293T cells. Cell lysates were immunoblotted with rabbit monoclonal anti-V5 antibody for NLRP12 and mouse monoclonal anti-HCK antibody. Comparing lane 2 (basal level of NLRP12 expression) with lane 7, co-expression of HCK protein leads to a significant decrease of protein expression levels of NLRP12. Increasing protein expression of HCK, relative to the plasmid DNA added (ramp: lanes 5, 6, and 7; 2.5-20 μg of DNA) was not observed possibly because the amount of plasmid for HCK transfection were already too high, resulting in the maximum expression of HCK at all plasmid DNA amounts. The non-increasing protein expression could also be due to saturation of the signal for HCK on the blot or a combination of the amount of plasmid for HCK transfection were already too high as well as saturation of the blot. The figure is representative of the results from three experiments. (B) Quantification of the NLRP12 protein bands (mean + standard error) over three times they are co-expressed to show that NLRP12 protein expression levels decreased in the presence of co-expressed HCK, relative to equal amounts of cell lysate being assayed. The NLRP12 protein expression levels are indicated as arbitrary units. (C) Quantification of the HCK protein bands (mean + standard error) over three times they are co-expressed confirming that HCK protein expression levels did not change although the amount of plasmid DNA for *HCK* increased. Figure (B) and (C) were generated by Prism 6.0 (GraphPad Software, Inc., San Diego, CA). Twenty μg of cell lysate proteins were loaded for each lane. ANOVA and Tukey tests were used to compare the NLRP12 and HCK protein bands.

Figure 6 shows that in 293T cells, the co-expression of increasing amount of HCK may led to a decrease in steady-state protein expression levels of NLRP12. Although, alternatively, the apparent decrease in expression level of NLRP12 might be due actually to a decrease in the epitope-tag itself or a decrease in the expression of the epitope-tagged protein only. Interestingly, HCK protein levels did not show a linearly increasing level of expression upon transfection with increasing amounts of plasmid DNA, possibly due to HCK's maximum expression levels in these cells being achieved with the lowest amounts of plasmid DNA, which may be resolved if the transfections are performed with lower amounts of plasmid DNA. This lack of linearity in protein expression could also be due to saturation of the signal for HCK on the blot at relatively low levels of HCK expression or a combination of HCK's maximum expression levels has been reached as well as saturation of the blot, as mentioned above. Alternatively, reduction of NLRP12 expression could be due to plasmid competition; that is, since the expression of both genes from either plasmid are driven from the same promoter, an increase in the amount of one plasmid may competitively decrease expression of the gene from the other plasmid due to their competition for transcription factors 35.

### In blood and marrow samples from patients with AML, *NLRP12* and *HCK* co-occur and are co-expressed

The odds ratio test (a statistical test that defines the likelihood of association of two events) determines whether two proteins either co-occur or are mutually exclusive in their expression. The Fisher exact test is a statistical test that uses P-values to determine if two events, such as co-occurrence or mutual exclusivity, are quantitatively significantly associated. Applying these tests to The Cancer Genome Atlas (TCGA) provisional data sets for AML patients 36-38, *NLRP12* and *NLRP1* are the only two *NLRP* family members that show a statistically significant co-occurrence with *HCK* (Table 2). However, *NLRP1*'s co-occurrence has a P value of 0.048, while* NLRP12*'s co-occurrence has a P value of <0.001. Therefore, *NLRP12* is most likely the only NLRP family mRNAs that shows co-occurrence with *HCK* in samples from patients with AML.

**Figure 7:**
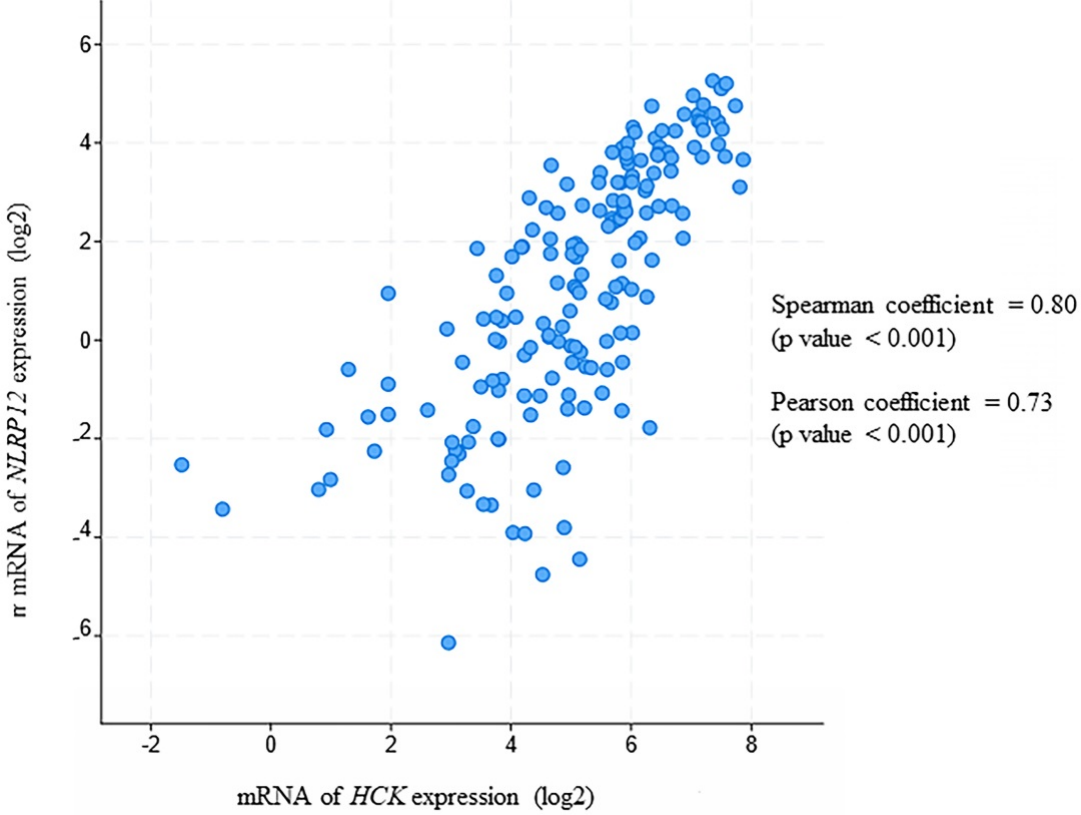
*NLRP12* is co-expressed with *Hck* in acute myeloid leukemia (AML) patient samples. Both *NLRP12* expression and *Hck* expression data are shown as log2 values. The data are RNA seq data obtained from cBioportal (provisional) database assessed on July 11, 2019. The graph was taken from the cBioportal website. Spearman coefficient, indicating the association of *NLRP12* and *Hck* mRNA expression level (if *NLRP12* and *Hck* mRNA expression levels are non-parametric) = 0.80. Pearson coefficient, indicating the degree of the linear relationship between the *NLRP12* mRNA expression level and *Hck* mRNA expression level (if *NLRP12* and *Hck* mRNA expression levels are parametric) = 0.73. The p-values for Spearman coefficient and Pearson coefficient are both < 0.001.

Moreover, in this analysis, *NLRP12* is significantly co-expressed with *HCK* (Pearson coefficient = 0.80 and Spearman coefficient = 0.73, Pearson and Spearman coefficients show the possibility of a linear relationship of two factors, Figure 7). This analysis is also consistent with *NLRP12* and *HCK* co-occurring in the same patients with AML. However, *NLRP12* was not the only *NLRP* mRNAs that shows significant co-expression with *HCK*. *NLRP1* and *NLRP3* also showed significant co-expression with *HCK*. This can be explained by the fact that co-expression is less stringent than co-occurrence. In summary, *NLRP12* is the only *NLRP* mRNAs that shows co-occurrence with *HCK*, and it also shows co-expression with *HCK*, although it is not the only member of the *NLRP* mRNAs to do so. Other data sets, including Hausera data, Metzeler 1 data, Metzeler2 data, and Raponi data, were not available to evaluate the co-expression of *NLRP12* versus *HCK*, as these other data sets did not incorporate data regarding *NLRP12* expression.

### Other potential binding partners of NLRP12 from the yeast two-hybrid screen

**Figure 8:**
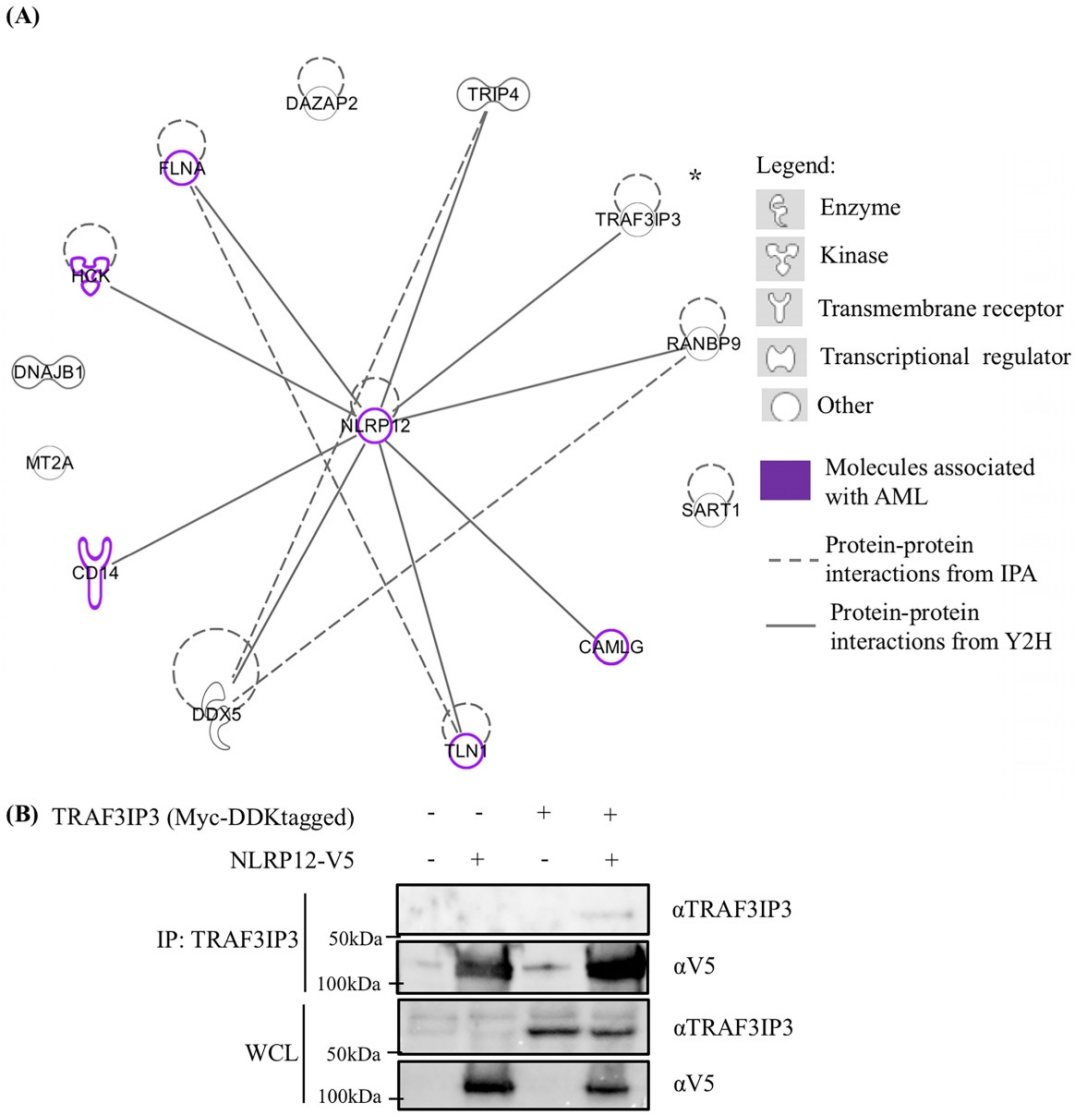
(A) Ingenuity Pathway Analysis (IPA) displaying the 13 distinctive hits from the total results of the yeast two-hybrid screen for proteins that interacted with NLRP12. The figure only shows the NLRP12-interacting proteins with hits that have a frequency larger than 2. The solid lines indicate interacting proteins found through our yeast two-hybrid screen. The dashed lines are the NLRP12-interacting proteins previously reported in published data. The purple-colored molecules are those associated with AML. (B) To validate an interaction shown in the IPA diagram, the Myc and DDK tagged form of tumor necrosis factor receptor-associated factor 3 interacting protein 3 (TRAF3IP3) and NLRP12-V5 were co-immunoprecipitated from co-transfected 293T cells. A non-denaturing cell lysate buffer (see Material and Methods) was used to lyse the cell, and co-immunoprecipitations were performed with a mouse monoclonal anti-TRAF3IP3 antibody followed by protein A/G agarose. The immunoprecipitates were analyzed for NLRP12 by immunoblotting with rabbit monoclonal anti-V5 antibody. The results from the co-immunoprecipitation are consistent with a confirmation of an interaction between TRAF3IP3 and NLRP12.

Other potentially interesting and unique genes that whose products may interact with NLRP12's PYD + NBD were identified in the yeast two-hybrid screen (Table 1 and Supplementary materials, Table S1). Table 1 only shows those hits with frequency larger than 2, and these hits were analyzed through an Ingenuity Pathway Analysis (IPA) diagram (Figure 8). In the IPA diagram, protein-protein interactions found by our yeast two-hybrid screen are connected by solid lines and interactions between protein binding partners collated by IPA are connected by dashed lines. In addition, those molecules that are associated with AML are colored purple. Interestingly, NLRP12 is also associated with AML from the database Catalogue Of Somatic Mutations In Cancer (COSMIC). Although the mutation occurs in the NBD of NLRP12, only 2 samples of these mutations are observed within 175 samples and may be worthy of follow-up study.

The interaction of one of the putative binding partners to NLRP12 identified in our screen, TRAF3 interacting protein 3 (TRAF3IP3), was validated in a co-immunoprecipitation experiment where the Myc and DDK tagged form of TRAF3IP3 and V5-tagged form of NLRP12 were overexpressed in 293T cells. TRAF3IP3 was coimmunoprecipitated by adding mouse monoclonal anti-TRAF3IP3 antibody, and a band that showed the same molecular weight size of NLRP12 was detected by rabbit monoclonal anti-V5 antibody (Figure 8). However, further validation of these other putative interactors detected by the yeast two-hybrid needs to be done.

## Discussion

In this paper, the yeast two-hybrid assay was used to screen for NLRP12 binding partners. This approach identified 48 genes as positive hits encoding proteins that could possibly bind to NLRP12. Among the hits, the c-SRC non-receptor tyrosine kinase family member HCK was selected to focus upon as a potentially interesting target, particularly given its association with hematopoiesis and AML. Further direct screens found that NLRP12 selectively binds to HCK, but not to NLRP8 or NLRP3. Conversely, HCK, but not other members of the c-SRC non-receptor tyrosine kinase family, selectively binds to NLRP12. HCK's C-terminal 30 amino acids are sufficient for binding to NLRP12. And, amino acids F503, I506, Q507, L510, and D511 of HCK are critical for binding to NLRP12. Co-immunoprecipitation experiments from mammalian cells confirmed that NLRP12 and HCK interact. Co-expression of NLRP12 and HCK results in a significantly lower steady-state expression level of NLRP12; thus, the interaction may affect NLRP12 protein stability. Finally, a bioinformatics analysis shows that *NLRP12* and *HCK* essentially exclusively co-occur, and mRNA levels for both are positively correlated in blood samples from AML patients. Together, these data suggested that NLRP12 and HCK protein and mRNA co-expression may have an effect on the AML.

The binding data from yeast two-hybrid (Figure 3 and Figure 4) showed that the R2 and R3 regions are both important for binding, but we suspect that R3 region is the major region that is critical for binding, while R2 region may be an “auxiliary” region that is also critical for binding, because when we delete the R2 region but maintain the R3 region, the R3 region itself cannot maintain stable binding of HCK's C-terminal 40 amino acids to NLRP12's PYD + NBD. Thus, binding of NLRP12's PYD + NBD to this fragment of HCK may occur initially through binding to R3, followed by stabilization of the interaction by secondarily binding to R2. This model is also consistent with the finding that when we mutated the R3 region, the binding of HCK's C-terminal 40 amino acid fragment to the NLRP12's PYD + NBD was lost despite the R2 region being present. The above evidences are consistent with the model that R2 and R3 regions together are both necessary for binding, but the stabilization of binding to the R3 region may require the presence of R2.

The yeast two-hybrid screen with NLRP12 also identified products from 47 other genes as potential interactors. According to the Search Tool for Recurring Instances of Neighbouring Genes (STRING) protein network and previous publications, NLRP12 interactors include IRAK1 39, NIK 34, TRAF3^3^, heat shock protein 90 (HSP90) 40, Fas associated factor 1 (FAF1) 41, and apoptosis- associated speck-like protein containing a caspase activation and recruitment domain (ASC) proteins. IRAK1, NIK, TRAF3, and Heat shock protein (HSP) 90 were only found in experiments involving semi-endogenous protein co-immunoprecipitations with NLRP12. FAF1 was found to be an interacting protein in NMR experiments by showing a difference in the chemical shift perturbation of FAF1's ubiquitin-associated domains (UBA) domain alone and NLRP12's PYD domain bound to FAF1's UBA domain. And FAF1 was also found to interact with NLRP12 in a yeast two-hybrid study where the PYD domain of different NLRP proteins were screened 42. However, in our study, IRAK1 43, NIK 44, TRAF3 45, HSP90 46, FAF1 47, and ASC 48 proteins were not found in our results of interacting proteins, although they are all expressed in leukocytes. Therefore, given these differences, the results from this yeast two-hybrid screen may provide a unique database for future investigations on the proteins that interact with NLRP12, and the functional outcomes of these interactions.

How HCK co-expression affects NLRP12 expression can be further investigated to determine the functional outcomes of the NLRP12-HCK interaction relative to protein stability or degradation. For example, MG-132 and chloroquine, which are a proteasomal inhibitor and an inhibitor of lysosomal degradation, respectively, can be added to cells in these experiments to determine whether the decreasing protein expression level of NLRP12 in the presence of HCK is due to proteasomal degradation or lysosomal degradation. In addition, an NLRP12 stable cell line, with a typically lower level of NLRP12 expression, compared to transiently transfected cells, can be used to test formally whether NLRP12 protein expression levels are increasingly decreased when the expression of HCK is increased. Moreover, the rate of NLRP12 degradation, in the absence or presence of HCK, can be studied and compared in pulse-chase assays 34.

Given the interaction that was characterized between NLRP12 and HCK, we sought to identify diseases in which their interaction may be relevant. AML is a type of blood and bone marrow cancer that is characterized by an unusually high level of circulating immature white blood cells 49. HCK is found to be highly expressed in hematopoietic stem cells of AML patients 24. The overexpression of HCK is linked with the development of AML 24,50. Therefore, HCK may be potentially used as a blood biomarker to identify those patients with potential to develop of AML disease. In addition, activation of HCK and its signaling pathways often causes the development of AML 25,51. HCK has been reported to be involved in the feline McDonough sarcoma (fms)-like tyrosine kinase 3 (FLT3)-HCK- cyclin D-dependent kinase (CDK6) pathway. The mutation in FLT3-internal tandem duplications (FLT3-ITD) causes the activation of HCK, and subsequently results in overexpression of CDK6 in MV4-11 cells, a human AML cell line with the FLT3-ITD mutation. *CDK6* siRNA resulted in MV4-11 cells with the FLT3-ITD mutation having a lower proliferation rate compared to MV4-11 control cells 25. Also, it has been reported that in AML patients, HCK, LYN, and FGR phosphorylation levels are high 51. In summary, the above evidence suggests that either high expression and/or high activation of HCK could occur in AML patients as well.

Our bioinformatics analysis revealed that *NLRP12* is likely the only member of the *NLRP* family that shows significant co-occurrence with *HCK* at the level of mRNA expression. However, our cellular functional study revealed that NLRP12 protein expression levels decreased when it is co-expressed with HCK. This discrepancy might be due to *NLRP12* mRNA expression level not correlating with protein expression level 52-54. Nevertheless, these data are still consistent with the possibility that NLRP12 may have an effect on AML through its binding to HCK. *NLRP12* and *HCK* co-expression and co-occurrence may then also be relevant to the pathogenesis of AML. However, further analyses need to be performed to evaluate the ability of NLRP12 and HCK to serve as biomarkers in the pathogenesis of AML.

*NLRP12* and *HCK* co-expression and co-occurrence may indicate that *NLRP12* and *HCK* mRNA or protein expression can impact the same pathway or both be regulated by the same molecule. For example, in the case two molecules exhibiting co-occurrence, they may interact in the same signaling pathway, for example, Ki-ras2 Kirsten rat sarcoma viral oncogene homolog (KRAS) and glycogen synthase kinase 3 beta (GSK3β) showed co-occurrence in the AML provisional data, and they are in the same signaling pathway 55.

Although many HCK inhibitors have been developed, they often lack specificity, with HCK inhibitors also inhibiting other members of the c-SRC non-receptor tyrosine kinase family 24,56-59. If the potential for the development of AML can be decreased when NLRP12 does not bind to HCK, then an inhibitor to the interaction between NLRP12 and HCK can be designed. On the other hand, if the potential for AML is increased when NLRP12 and HCK does not bind, then a drug that facilitates NLRP12 and HCK interaction could be developed. By this approach, the non-specificity problem with NLRP inhibitors may still exist but perhaps to a smaller extent, as NLRP proteins are less conserved overall compared to the members of the c-SRC non-receptor tyrosine kinase family. For example, the amino acid identity between NLRP12's PYD + NBD versus another NLRP PYD + NBD most closely related to NLRP12, NLRP3's PYD + NBD, is 54%. But, the protein identity between the HCK C1 fragment versus the one with the closest relationship, the LYN C1 fragment, is 78%.

In conclusion, this paper reports the preliminary findings of NLRP12 interacting with HCK, and *NLRP12* co-occurs with *HCK* in AML patients. The binding data presented here will expand the database regarding the interactors of NLRP12, and additional bioinformatic and functional *in vitro* and *in vivo* analyses may shed light on the functional consequence of the interaction of NLRP12 with HCK.

## Material and Methods

### Reagents and cells

U937, THP-1, RAW 264.7, and 293T cells were all purchased from American Type Culture Collection (ATCC) (Manassas, VA). pCDNA-*NLRP12* plasmid and pCDNA-*NLRP3* plasmid were gifts from Dr. Jenny P. Y. Ting (University of North Carolina-Chapel Hill). pCDNA-*NLRP8* plasmid was purchased from Genescript (Piscataway, NJ). pCMV6-*TRAF3IP3* (Myc-DDK tagged) was purchased from OriGene (Rockville, MD). All other chemicals were reagent grade. The mouse monoclonal anti-FLAG antibody and the rabbit monoclonal anti-FLAG antibody were purchased from Sigma-Aldrich (St. Louis, MO). The mouse monoclonal anti-HCK antibody, the mouse monoclonal anti-TRAF3IP3 antibody, the mouse monoclonal anti-IgG antibody, and the mouse IgGκ binding protein-horseradish peroxidase (HRP) were purchased from Santa Cruz Biotechnology (Dallas, TX). The mouse monoclonal anti-V5 antibody, the rabbit monoclonal anti-V5 antibody, the β-actin rabbit mAb (HRP conjugate), the horse anti-mouse IgG HRP-linked secondary antibody, and the goat anti-rabbit IgG HRP-linked secondary antibody were purchased from Cell Signaling (Beverly, MA). The Clean-Blot™ IP Detection Reagent was purchased from Thermo Fisher Scientific (Grant Island, NY). Goat anti-mouse IgG (H+L) cross-adsorbed secondary antibody, conjugated to Alexa Fluor 488 and goat anti-rabbit IgG (H+L) cross-adsorbed secondary antibody, conjugated to Alexa Fluor 555 were purchased from Thermo Fisher Scientific. The 4',6- diamidino-2-phenylindole, dihydrochloride (DAPI) was purchased from Thermo Fisher Scientific.

### Cloning

*NLRP12*'s PYD + NBD (corresponding to a.a. 1-531), amplified by polymerase chain reaction (PCR) (primers see Table 3) from a pCDNA-*NLRP12* plasmid, was digested with enzymes NdeI and SalI, and subcloned into the pGBKT7 vector (Takara). The sequence encoding the PYD + NBD of *NLRP3* (corresponding to a.a. 1-536) was amplified by PCR (see table 3 for primers), digested with enzymes NdeI and BamHI, and subcloned in the pGBKT7 vector. The sequence encoding the PYD + NBD of *NLRP8* (corresponding to a.a. 1- 527) was amplified by PCR (see Table 3 for primers), digested with enzymes EcoRI and BamHI, and subcloned into the pGBKT7 vector. The entire sequences corresponding to the C-terminal 42 amino acids of FGR, c-SRC, YES, and FYN, and 40 amino acids of BLK, LCK, LYN, and HCK (The C terminal 40 (or 42) amino acids is called “C1”), HCK constructs C2, C3, C4, C5, C6, C7, N1, N2, N3, N4, N5, M1, M2, M3, M4, and HCK C1 mutations for alanine scanning with F503A, E504A, Y505A, I506A, Q507A, S508A, V509A, L510A, and D511A, were all synthesized from IDT DNA (Coralville, IA) and cloned into the pACT2 vector (Takara). *NLRP12* was amplified by PCR (see Table 3 for primers), digested with AflII and XbaI, and subcloned into pEFIRES-P-3FLAG 60 and pEFIRES -P-V5 60. 3FLAG and V5 are homemade, i.e., added later into pEFIRES-P. The peptide sequence of the 3FLAG epitope is DYKDDDDKDYKDDDDKDYKDDDDK. The peptide sequence of the V5 epitope is GKPIPNPLLGLDST. The multiple cloning sites of pEFIRES-P were modified according to our needs. Sequences encoding HCK p61 and p59 isoforms were amplified by PCR (see Table 3 for primers), digested with AflII and XbaI, and subcloned into pEFIRES -3FLAG vector. Final plasmids were sequenced to confirm the constructs.

### Yeast two-hybrid assay

#### Yeast transformed with the plasmid PGBKT7- NLRP12's PYD + NBD

Yeast strain AH109 (Takara, Mountain View, CA) was stored frozen at -80°C. Upon use, it was streaked onto an agar plate supplemented with yeast extract, peptone, dextrose, and adenine hemisulfate (YPDA) (Takara), which was then placed in a 30°C incubator overnight. The growing colonies were picked and placed into 10 ml of YPDA media, incubated overnight with shaking at 30°C. Then they were transformed with *NLRP12*'s PYD + NBD plasmid, following recommendations in the Takara manual (Matchmaker^TM^ Gal4 Two-hybrid System 3 and Libraries User Manual). A 4ml of 1 M of 3-AT was added into 800 mL of the YPDA medium that were used for making the plates due to a leakage problem. 3-AT is a competitive inhibitor for the product of the *HIS3* reporter gene. Yeast cells were lysed using a trichloroacetic acid (TCA) method following the instructions in the Takara manual (Yeast protocol handbook), and western blots were performed to check NLRP12's PYD + NBD protein expression (data not shown).

#### Library scale transformation of cDNA library into yeast that have been transformed with the PGBKT7- NLRP12 PYD + NBD plasmid

A human leukocyte Matchmaker^TM^ cDNA library was purchased from Takara. The library was titered according to the Matchmaker^TM^ Gal4 Two-hybrid System 3 and Libraries User Manual. The colony-forming units (CFU) for the library were calculated to be about 3.1 x 10^9^ CFU/ml. Four large scale transformations (equal to one library scale) were done at the same time following the Matchmaker^TM^ Gal4 Two-hybrid System 3 and Libraries User's Manual. To calculate the efficiency of yeast two-hybrid (i.e., the number of colons obtained per µg of library plasmid DNA) and to check the expressions of “bait” and “prey” plasmids, small amounts of the solution, diluted 10X, 100X, 1000X, and 10,000X were plated in SD/-Trp-Leu 2DO plates. The yeast two-hybrid efficiency obtained was 3 x 10^6^ CFU per µg of library plasmid DNA. In addition, if the yeast grew on the SD/-Trp-Leu-His-Ade + 3-AT plates, the “bait” and “prey” plasmids were considered to be interacting with each other.

#### Small scale transformation

The yeast two-hybrid co-transformation on a small scale for all of the direct screenings was performed following the small-scale transformation protocol in the Matchmaker^TM^ Gal4 Two-hybrid System 3 & Libraries User's Manual. The direct screens were to test whether NLRP12 PYD + NBD and HCK selectively interact; identification of HCK F503, I506, Q507, L510, and D511 being critical for binding with NLRP12 PYD + NBD; and the characterization of the HCK C-terminal 30 amino acid fragment is the shortest fragment that binds to NLRP12 PYD + NBD.

### Cell transfection

293T cells, THP-1 cells, U937 cells, and RAW 264.7 cells were seeded at a density of 5 x 10^5^ per well in a 6-well plate one day before transfection. Transfection followed the Lipofectamine® 3000 reagent (Thermo Fisher Scientific) protocol with the following modifications. Briefly, 3.75 µl of Lipofectamine® 3000 was added into 125 µl of Opti-MEM® medium (Thermo Fisher Scientific). 2.5 µg of plasmid DNA (prepared by isolation through cesium chloride (CsCl) density gradient purification 61 and 5 µl of P3000^TM^ reagent were added into another aliquot of 125 µl of Opti-MEM® medium. Then these two tubes were mixed together, and the mixed tube was incubated at room temperature for 15 min. 250 µl of this mixture was added to the cells. The cells were incubated at 37 °C with 5% CO_2_ for two days until analysis.

### NLRP12 stable cell lines

THP-1 cells, U937 cells, and RAW 264.7 cells were transfected with pEFIRES-P-*NLRP12*-3FLAG plasmid, and 2.5 μg/ml puromycin (Invitrogen^TM^ by Thermo Fisher Scientifc) was added. After two weeks, the cells were considered to be stably expressing NLRP12 after screening for expression. The stable cells were “pooled” clones, and they are maintained by adding 2.5 μg/ml puromycin.

### Cell lysis and immunoprecipitation

After trypsinization, cells were lysed in 500 μl of lysis buffer (1% NP-40, 4mM Tris-HCl, pH 8.0, 150 mM NaCl). Cells were then treated with freeze-thaw cycles, i.e., frozen in the -80ºC freezer for 5 min and thawed at 37ºC in a water bath for 3 min, for a total of five times. Cells were then centrifuged at 12,000 x g for 10 min at 4ºC. After centrifugation, the supernatant was saved at -20ºC until further use.

For immunoprecipitations, cell lysates were pre-cleared by adding 40 μl of protein A/G agarose beads (Thermo Fisher Scientific) from a 50% slurry, and the sample was rotated for 1 hr at 4ºC. The sample was spun at 1000 x g for 1min. Then, 30 μl of pre-cleared cell lysate were removed and run on immunoblotting as a control. The remaining cell lysate was incubated with 1 μg mouse monoclonal anti-HCK primary antibody (1 μg mouse monoclonal anti-TRAF3IP3 primary antibody, or 1 μg mouse monoclonal anti-IgG primary antibody) overnight. On the second day, 30 μl of protein A/G agarose, from a 50% slurry and previously washed with lysis buffer three times, were added to the tube, and the tube was incubated for 3 hrs at 4ºC. The beads were then washed with lysis buffer, but with the NaCl concentration increased to 500 mM. The tube was briefly centrifuged at 1000 x g for 1min., and the supernatant was removed. This step was performed for a total of 5 times. The proteins were eluted from the beads by addition of SDS-PAGE sample buffer.

### Immunoblotting

Protein concentrations were determined by the Pierce^TM^ BCA protein assay (Thermo Fisher Scientific). About 20µg of cell lysate samples and immunoprecipitated samples were loaded onto the 8% acrylamide gel. After overnight transfer of the proteins from an SDS-gel to a 0.45 μm pore size polyvinylidene difluoride (PVDF) membrane (Thermo Fisher Scientific), the membrane was blocked in for 1 hr at room temperature in 5% non-fat dry milk dissolved in Tris-buffered saline with Tween 20, pH=8.0 (TBST) (Sigma-Aldrich). Mouse monoclonal anti-FLAG antibody (mouse monoclonal anti-V5 antibody, rabbit monoclonal anti-V5 antibody, the mouse monoclonal anti-TRAFIP3 antibody, or mouse monoclonal anti-IgG antibody) (diluted 1:1000) and mouse monoclonal anti-HCK antibody (diluted 1:1000) were added and the membrane incubated overnight. For detection of bands in the cell lysate, rabbit anti-mouse IgG HRP-linked secondary antibody (diluted 1:2000) (for anti-FLAG antibody, anti-V5 antibody, anti-TRAF3IP3 antibody, and anti-IgG antibody), goat anti-rabbit IgG HRP-linked secondary antibody (for anti-V5 antibody) (diluted 1:3000), and mouse IgGκ binding protein-HRP secondary antibody (for anti-HCK antibody) (diluted 1:1000) were added, and the membrane was incubated for 1 hr at room temperature. For immunoprecipitations, Clean-Blot™ IP detection reagent (HRP) secondary antibody (1:300) (Thermo Fisher Scientific) was used. The detection signal was visualized by using enhanced chemiluminescence (ECL) reagent (Thomas Scientific, Swedesboro, NJ). Immoblots were analyzed by chemiluminescence on a Bio-Rad ChemiDoc^TM^ Touch Gel imaging system (Bio-Rad, Hercules, CA).

### Immunofluorescence

The coverslips were autoclaved and coated with 0.1 mg/ml of poly-D-lysine hydrobromide (mol wt 70,000-150,000) (Sigma-Aldrich). Cells were plated onto the coverslips. The cells were fixed in 4% paraformaldehyde (Alfa Aesar, Ward Hill, MA) in phosphate buffered saline (PBS) for 15 mins. And fixation was stopped by adding 50 mM NH_4_Cl in PBS for 15 min at room temperature. After raising with PBS, the fixed cells were permeabilized by adding 0.5% Triton®-X-100 in PBS for 10 min. The cells were then washed with PBS three times and blocked with 0.1% of bovine serum albumin (BSA) in PBS at room temperature for 1 hr. The rabbit monoclonal anti-FLAG antibody (diluted 1:100) and mouse monoclonal anti-HCK antibody (diluted 1:100) were added to each cover slip and incubated overnight. Goat anti-mouse IgG (H+L) cross-adsorbed secondary antibody, conjugated to Alexa Fluor 488 (diluted 1:100) and goat anti-rabbit IgG (H+L) cross-adsorbed secondary antibody, conjugated to Alexa Fluor 555 (1: 100) were added. After incubation with secondary antibodies for 1 hr, the cells were washed five times with PBS. During the first time of washing, DAPI (Invitrogen^TM^ by Thermo Fisher Scientific) was also added at 1:1000 in PBS for 2 min. Finally, the coverslips were mounted in ProLong™ Gold Antifade mounting medium (Life Technologies Corporation by Thermo Fisher Scientific, Eugene, OR). The stained slides were viewed on a ZEISS LSM 880 (Pleasanton, CA) with Airyscan. The images were viewed and processed using Fiji software 62.

### IPA diagram

Yeast two-hybrid data were analyzed by IPA software (QIAGEN Inc., https://www.qiagenbioinformatics.com/products/ingenuity- pathway-analysis).

### Statistical analysis

The Spearman coefficient and Pearson coefficient used for co-expression was generated by cBioPortal website 37,38,63. Clinical samples from the cBioportable for cancer genomics (https://www.cbioportal.org) and Oncomine (https://www.oncomine.org) were downloaded and checked for *NLRP12* and *Hck* mRNA expression levels. The Fisher exact test and the odds ratio test used for co-occurrence were generated by the cBioPortal website. Analysis of variance (ANOVA) was performed to check whether a significant difference of the treatment exists among all the groups. A p-value < 0.05 was considered statistically significant. If P < 0.05, then the Tukey test was used to determine whether a pair-wise difference exists. Quantification of the western blot bands was performed by FIJI software 62 and made into graphs by Prism 6.0 (GraphPad Software, Inc., San Diego, CA).

## Supplementary Material

Supplementary figure and table.

## Figures and Tables

**Table 1:** yeast two hybrid results (hit frequency >= 3).

Name	Frequency
Deleted in azoospermia (DAZ) associated protein 2 (DAZAP2)	10
Filamin A, alpha (FLNA)	9
Hematopoietic cell kinase (HCK) proto-oncogene, c-SRC family tyrosine kinase (HCK)	9
Chaperone DnaJ (a.k.a., heat shock protein 40 kD(Hsp40)) homolog, subfamily B, member 1 (DNAJB1)	9
Metallothionein 2A (MT2A)	7
Cluster of differentiation 14 molecule (CD14)	6
DEAD (Asp-Glu-Ala-Asp) box helicase 5 (DDX5)	6
Talin 1 (TLN1)	5
Calcium modulating ligand (CAMLG)	4
Squamous cell carcinoma antigen recognized by T cells (SART1)	4
Ras-related nuclear protein (RAN) binding protein 9 (RANBP9)	3
Tumor necrosis factor (TNF) receptor associated factor 3 (TRAF3) interacting protein 3 (TRAF3IP3)	3
Thyroid hormone receptor interactor 4 (TRIP4)	3

**Table 2:** Co-occurrence of mRNA expression level of *HCK* and all of the *NLRP* family proteins (from the provisional data, assessed on July 11 2019).

*HCK* + *NLRP* mRNAs	Co-occurrence (C) or mutual exclusivity (M)	P-value
*NLRP*1	C	0.048
*NLRP*2	M	0.546
*NLRP*3	C	0.415
*NLRP*4	M	0.936
*NLRP*5	M	0.877
*NLRP*6	M	0.670
*NLRP*7	M	0.475
*NLRP*8	M	0.877
*NLRP*9	M	0.491
*NLRP*10	M	0.626
*NLRP*11	M	0.233
*NLRP*12	C	<0.001
*NLRP*13	C	1.000
*NLRP*14	M	0.509

**Table 3:** Primers used in cloning (The underline shows the restriction enzymes).

Name	Primers
*NLRP12* (a.a. 1-531) 5' primer (cloned into PGBKT7)	GATCTACATATGCTACGAACCGCAGGCAG
*NLRP12* (a.a. 1-531) 3' primer (cloned into PGBKT7)	GATGTAGTCGACTCACCCCTCGTCCAGGATATAGTACATAGCT
*NLRP3* (a.a. 1-536) 5' primer (cloned into pGBKT7)	GATCTACATATGAAGATGGCAAGCACCCGCTGCA
*NLRP3* (a.a. 1-536) 3' primer (cloned into PGBKT7)	GATGTAGGATCCTCACAGCAGGTAGTACATGGCGGCAAAGA
*NLRP8* (a.a. 1-527) 5' primer (cloned into pGBKT7)	GATCTAGAATTCAGTGACGTGAATCCACCCTCTG
*NLRP8* (a.a. 1-527) 3' primer (cloned into PGBKT7)	GATGTAGGATCCTCAGAGTCTTTGTGGGAAACAGAGAACA
*NLRP12* 5' (cloned into pEFIRES-P-3FLAG and pIRES-V5)	GTCCAGCTTAAGGCCACCATGCTACGAACCGCAGGCAG
*NLRP12* 3' (cloned into PIRES-3FLAG and pEFIRES-P-V5)	CTGAATTCTAGAGCAGCCAATGTCCAAATAAGG
*HCK p61* 5' (cloned into pEFIRES-P-3FLAG)	GATCTACTTAAGGCCACCATGGGGGGGCGCTCAAGCTGCGAGG
*HCK p61/p59* 3' (cloned into pEFIRES-P-3FLAG)	GTCGATTCTAGATGGCTGCTGTTGGTACTGGCTCTCT

## Abbreviations

3-AT: 3-Amino-1,2,4-triazole
AML: acute myeloid leukemia
ANOVA: analysis of variance
ASC: apoptosis-associated speck-like protein containing a caspase activation and recruitment domain
ATCC: American Type Culture Collection
C1: self-named truncations of C-terminal 40 (or 42) amino acids fragment of c-SRC family non-receptor tyrosine kinases
C2-C7 and N1-N5: self-named truncations HCK C1 fragment
CD40: cluster of differentiation 40
CDK6: cyclin D-dependent kinase 6
CFU: colony-forming units
COSMIC: catalogue of somatic mutations in cancer
CsCl: cesium chloride
DAMP: damage-associated molecular patterns
DAPI: 4',6-diamidino-2-phenylindole, dihydrochloride
DC: dendritic cells
DO: drop out (2DO: SD/-Trp-Leu
4DO: SD/-Trp-Leu-His-Ade)
EAE: experimental autoimmune encephalomyelitis
ECL: enhanced chemiluminescence
FAF1: Fas associated factor 1
FLT3-ITD: FLT3-internal tandem duplications
FLT3: feline McDonough sarcoma (fms)-like tyrosine kinase 3
GSK3β: glycogen synthase kinase 3 beta
HCK C1 fragment: HCK C-terminal 40 amino acids
HCK: hematopoiesis cell kinase
HRP: horseradish peroxidase
HSP90: heat shock protein 90
IL: interleukin
IPA: ingenuity pathway analysis
IRAK1: IL-1 receptor-associated kinase 1
c-JNK: c-Jun N-terminal kinase
KO: knock out
KRAS: Ki-ras2 Kirsten rat sarcoma viral oncogene homolog
LRR: leucine rich repeat domain
M1: the amino acids in the R1 region were mutated to the amino acids identical to the R1 region in Lyn
M2: the amino acids in the R2 region were all mutated to Alanine
M3: the amino acids in the R3 region were all mutated to Alanine
M4: the amino acids in the R4 region were all mutated to Alanine
NBD: nucleotide binding domain
NF-κB: nuclear factor kappa-light-chain-enhancer of activated B cells
NIK: NF-κB-inducing kinase
NLR: nucleotide binding domain and leucine-rich repeat receptor
NLRP: NLR pyrin domain-containing protein
PAMP: pathogen-associated molecular patterns
PBS: phosphate buffered saline
PCR: polymerase chain reaction
PDB: protein data bank
PMA: phorbol 12-myristate 13-acetate
PRR: pattern recognition receptors
PVDF: polyvinylidene difluoride
PYD: pyrin domain
R1, R2, R3, and R4: self-named four regions in the HCK C terminal 40 amino acids fragment
SD: synthetic dropout
SD/-Trp-Leu: SD base plus amino acids supplements minus tryptophan and leucine
SD/-Trp-Leu-His- Ade: SD base plus amino acids supplements minus tryptophan, leucine, and histidine, and adenine
STRING: search tool for recurring instances of neighboring genes
TBST: tris-buffered saline with Tween 20, pH=8.0
TCA: trichloroacetic acid
TCGA: the cancer genome atlas
TLR: toll-like receptor
TRAF3: tumor necrosis factor receptor associated factor 3
TRAF3IP3: TRAF3 interacting protein 3
UBA: ubiquitin-associated domains
WT: wild type
YPDA: yeast extract, peptone, dextrose, and adenine hemisulfate

## References

[1] Takeuchi O, Akira S (2010). Pattern recognition receptors and inflammation. Cell.

[2] Tuncer S, Fiorillo MT, Sorrentino R (2014). The multifaceted nature of NLRP12. J Leukoc Biol.

[3] Allen IC, Wilson JE, Schneider M, Lich JD, Roberts RA, Arthur JC, Woodford RM, Davis BK, Uronis JM, Herfarth HH, Jobin C, Rogers AB, Ting JP (2012). NLRP12 suppresses colon inflammation and tumorigenesis through the negative regulation of noncanonical NF-kappaB signaling. Immunity.

[4] Zaki MH, Vogel P, Malireddi RK, Body-Malapel M, Anand PK, Bertin J, Green DR, Lamkanfi M, Kanneganti TD (2011). The NOD-like receptor NLRP12 attenuates colon inflammation and tumorigenesis. Cancer Cell.

[5] Chen ST, Chen L, Lin DS, Chen SY, Tsao YP, Guo H, Li FJ, Tseng WT, Tam JW, Chao CW, Brickey WJ, Dzhagalov I, Song MJ, Kang HR, Jung JU, Ting JP (2019). NLRP12 regulates anti-viral RIG-I activation via interaction with TRIM25. Cell Host Microbe.

[6] Chen L, Wilson JE, Koenigsknecht MJ, Chou WC, Montgomery SA, Truax AD, Brickey WJ, Packey CD, Maharshak N, Matsushima GK, Plevy SE, Young VB, Sartor RB, Ting JP (2017). NLRP12 attenuates colon inflammation by maintaining colonic microbial diversity and promoting protective commensal bacterial growth. Nat Immunol.

[7] Truax AD, Chen L, Tam JW, Cheng N, Guo H, Koblansky AA, Chou WC, Wilson JE, Brickey WJ, Petrucelli A, Liu R, Cooper DE, Koenigsknecht MJ, Young VB, Netea MG, Stienstra R, Sartor RB, Montgomery SA, Coleman RA, Ting JP (2018). The Inhibitory Innate Immune Sensor NLRP12 maintains a threshold against obesity by regulating gut microbiota homeostasis. Cell Host Microbe.

[8] Ye Z, Lich JD Moore CB, Duncan JA Williams KL, Ting JP (2008). ATP binding by monarch-1/NLRP12 is critical for its inhibitory function. Mol Cell Biol.

[9] Zaki MH, Man SM, Vogel P, Lamkanfi M, Kanneganti TD (2014). Salmonella exploits NLRP12-dependent innate immune signaling to suppress host defenses during infection. Proc Natl Acad Sci U S A.

[10] Jeru I, Le Borgne G, Cochet E, Hayrapetyan H, Duquesnoy P, Grateau G, Morali A, Sarkisian T, Amselem S (2011). Identification and functional consequences of a recurrent NLRP12 missense mutation in periodic fever syndromes. Arthritis Rheum.

[11] Jeru I, Duquesnoy P, Fernandes-Alnemri T, Cochet E, Yu JW, Lackmy-Port-Lis M, Grimprel E, Landman-Parker J, Hentgen V, Marlin S, McElreavey K, Sarkisian T, Grateau G, Alnemri ES, Amselem S (2008). Mutations in NALP12 cause hereditary periodic fever syndromes. Proc Natl Acad Sci U S A.

[12] Arthur J C, Lich JD, Ye Z, Allen IC, Gris D, Wilson JE, Schneider M, Roney KE, O'Connor BP, Moore CB, Morrison A, Sutterwala FS, Bertin J, Koller BH, Liu Z, Ting JP (2010). Cutting edge: NLRP12 controls dendritic and myeloid cell migration to affect contact hypersensitivity. J Immunol.

[13] Lukens JR, Gurung P, Shaw PJ, Barr MJ, Zaki MH, Brown SA, Vogel P, Chi H, Kanneganti TD (2015). The NLRP12 sensor negatively regulates autoinflammatory disease by modulating interleukin-4 production in T cells. Immunity.

[14] Vladimer GI, Weng D, Paquette SWM, Vanaja SK, Rathinam VA, Aune MH, Conlon J E, Burbage JJ, Proulx MK, Liu Q (2012). The NLRP12 inflammasome recognizes *Yersinia pestis*. Immunity.

[15] Udden SN, Kwak YT, Godfrey V, Khan MAW, Khan S, Loof N, Peng L, Zhu H, Zaki H (2019). NLRP12 suppresses hepatocellular carcinoma via downregulation of cJun N-terminal kinase activation in the hepatocyte. Elife.

[16] Hornick EE, Banoth B, Miller AM, Zacharias ZR, Jain N, Wilson ME, Gibson-Corley KN, Legge KL, Bishop GA, Sutterwala FS, Cassel SL (2018). NLRP12 mediates adverse neutrophil recruitment during influenza virus infection. J Immunol.

[17] Damiano JS, Oliveira V, Welsh K, Reed JC (2004). Heterotypic interactions among NACHT domains: implications for regulation of innate immune responses. The Biochemical journal.

[18] Janowski AM, Kolb R, Zhang W, Sutterwala FS (2013). Beneficial and detrimental roles of NLRs in carcinogenesis. Frontiers in immunology.

[19] Faustin B, Lartigue L, Bruey JM, Luciano F, Sergienko E, Bailly-Maitre B, Volkmann N, Hanein D, Rouiller I, Reed JC (2007). Reconstituted NALP1 inflammasome reveals two-step mechanism of caspase-1 activation. Mol Cell.

[20] Martinon F, Agostini L, Meylan E, Tschopp J (2004). Identification of bacterial muramyl dipeptide as activator of the NALP3/cryopyrin inflammasome. Curr Biol.

[21] Kanneganti TD, Lamkanfi M, Nunez G (2007). Intracellular NOD-like receptors in host defense and disease. Immunity.

[22] Sharif H, Wang L, Wang WL, Magupalli VG, Andreeva L, Qiao Q, Hauenstein AV, Wu Z, Núñez G, Mao Y, Wu H (2019). Structural mechanism for NEK7-licensed activation of NLRP3 inflammasome. Nature.

[23] Hafner-Bratkovič I, Sušjan P, Lainšček D, Tapia-Abellán A, Cerović K, Kadunc L, Angosto-Bazarra D, Pelegrin P, Jerala R (2018). NLRP3 lacking the leucine-rich repeat domain can be fully activated via the canonical inflammasome pathway. Nature Communications.

[24] Roversi FM, Pericole FV, Machado-Neto JA, da Silva Santos Duarte A, Longhini AL, Corrocher FA, Palodetto B, Ferro KP, Rosa RG, Baratti MO, Verjovski-Almeida S, Traina F, Molinari A, Botta M, Saad ST (2017). Hematopoietic cell kinase (HCK) is a potential therapeutic target for dysplastic and leukemic cells due to integration of erythropoietin/PI3K pathway and regulation of erythropoiesis: HCK in erythropoietin/PI3K pathway. Biochim Biophys Acta Mol Basis Dis.

[25] Lopez S, Voisset E, Tisserand JC, Mosca C, Prebet T, Santamaria D, Dubreuil P, De Sepulveda P (2016). An essential pathway links FLT3-ITD, HCK and CDK6 in acute myeloid leukemia. Oncotarget.

[26] Normand S, Waldschmitt N, Neerincx A, Martinez-Torres RJ, Chauvin C, Couturier-Maillard A, Boulard O, Cobret L, Awad F, Huot L, Ribeiro-Ribeiro A, Lautz K, Ruez R, Delacre M, Bondu C, Guilliams M, Scott C, Segal A, Amselem S, Hot D, Karabina S, Bohn E, Ryffel B, Poulin LF, Kufer TA, Chamaillard M (2018). Proteasomal degradation of NOD2 by NLRP12 in monocytes promotes bacterial tolerance and colonization by enteropathogens. Nature Communications.

[27] Linz BM, Neely CJ, Kartchner LB, Mendoza AE, Khoury AL, Truax A, Sempowski G, Eitas T, Brickey J, Ting JP, Cairns BA, Maile R (2017). Innate immune cell recovery is positively regulated by NLRP12 during emergency hematopoiesis. J Immunol.

[28] Robbins SM, Quintrell NA, Bishop JM (1995). Myristoylation and differential palmitoylation of the HCK protein-tyrosine kinases govern their attachment to membranes and association with caveolae. Mol Cell Biol.

[29] Carreno S, Gouze ME, Schaak S, Emorine LJ, Maridonneau-Parini I (2000). Lack of palmitoylation redirects p59HCK from the plasma membrane to p61HCK-positive lysosomes. J Biol Chem.

[30] Guiet R, Poincloux R, Castandet J, Marois L, Labrousse A, Le Cabec V, Maridonneau-Parini I (2008). Hematopoietic cell kinase (HCK) isoforms and phagocyte duties - from signaling and actin reorganization to migration and phagocytosis. Eur J Cell Biol.

[31] Sicheri F, Moarefi I, Kuriyan J (1997). Crystal structure of the SRC family tyrosine kinase HCK. Nature.

[32] Ma B, Elkayam T, Wolfson H, Nussinov R (2003). Protein-protein interactions: Structurally conserved residues distinguish between binding sites and exposed protein surfaces. Proc Natl Acad Sci U S A.

[33] Knappe M, Bodevin S, Selinka HC, Spillmann D, Streeck RE, Chen XS, Lindahl U, Sapp M (2007). Surface-exposed amino acid residues of HPV16 L1 protein mediating interaction with cell surface heparan sulfate. J Biol Chem.

[34] Lich JD, Williams KL, Moore CB, Arthur JC, Davis BK, Taxman DJ, Ting JP (2007). Monarch-1 suppresses non-canonical NF-kappaB activation and p52-dependent chemokine expression in monocytes. J Immunol.

[35] Lin M, Fu J, Liu S (2013). A repeat sequence causes competition of ColE-type plasmids. PLoS One.

[36] Cerami E, Gao J, Dogrusoz U, Gross BE, Sumer SO, Aksoy BA, Jacobsen A, Byrne CJ, Heuer ML, Larsson E, Antipin Y, Reva B, Goldberg AP, Sander C, Schultz N (2012). The cBio Cancer Genomics Portal: An open platform for exploring multidimensional cancer genomics data. Cancer Discovery.

[37] Gao J, Aksoy BA, Dogrusoz U, Dresdner G, Gross B, Sumer SO, Sun Y, Jacobsen A, Sinha R, Larsson E, Cerami E, Sander C, Schultz N (2013). Integrative analysis of complex cancer genomics and clinical profiles using the cBioPortal. Sci Signal.

[38] Cancer Genome Atlas Research Network (2013). Genomic and epigenomic landscapes of adult de novo acute myeloid leukemia. NEJM.

[39] Williams KL, Lich JD, Duncan JA, Reed W, Rallabhandi P, Moore C, Kurtz S, Coffield VM, Accavitti-Loper MA, Su L, Vogel SN, Braunstein M, Ting JP (2005). The CATERPILLER protein monarch-1 is an antagonist of toll-like receptor-, tumor necrosis factor alpha-, and Mycobacterium tuberculosis-induced pro-inflammatory signals. J Biol Chem.

[40] Arthur JC, Lich JD, Aziz RK, Kotb M, Ting JP (2007). Heat shock protein 90 associates with monarch-1 and regulates its ability to promote degradation of NF-kappaB-inducing kinase. J Immunol.

[41] Pinheiro AS, Eibl C, Ekman-Vural Z, Schwarzenbacher R, Peti W (2011). The NLRP12 pyrin domain: structure, dynamics, and functional insights. Journal of molecular biology.

[42] Kinoshita T, Kondoh C, Hasegawa M, Imamura R, Suda T (2006). Fas-associated factor 1 is a negative regulator of PYRIN-containing Apaf-1-like protein 1. Int Immunol.

[43] Shi C, Miley J, Nottingham A, Morooka T, Prosdocimo DA, Simon DI (2019). Leukocyte integrin signaling regulates FOXP1 gene expression via FOXP1-IT1 long non-coding RNA-mediated IRAK1 pathway. Biochim Biophys Acta Gene Regul Mech.

[44] Fu G, Xu Q, Qiu Y, Jin X, Xu T, Dong S, Wang J, Ke Y, Hu H, Cao X, Wang D, Cantor H, Gao X, Lu L (2017). Suppression of Th17 cell differentiation by misshapen/NIK-related kinase MINK1. J Exp Med.

[45] Wang Q, Liu F, Zhang M, Zhou P, Xu C, Li Y, Bian L, Liu Y, Yao Y, Wang F, Fang Y, Li D (2018). NLRP12 promotes mouse neutrophil differentiation through regulation of non-canonical NF-kappaB and MAPK(ERK1/2) signaling. Int J Biol Sci.

[46] Kinnaird JH, Singh M, Gillan V, Weir W, Calder ED, Hostettler I, Tatu U, Devaney E, Shiels BR (2017). Characterization of HSP90 isoforms in transformed bovine leukocytes infected with *Theileria annulata*. Cell Microbiol.

[47] Ryu SW, Lee SJ, Park MY, Jun JI, Jung YK, Kim E (2003). Fas-associated factor 1, FAF1, is a member of Fas death-inducing signaling complex. J Biol Chem.

[48] Martin BN, Wang C, Zhang CJ, Kang Z, Gulen MF, Zepp JA, Zhao J, Bian G, Do JS, Min B, Pavicic PG Jr, El-Sanadi C, Fox PL, Akitsu A, Iwakura Y, Sarkar A, Wewers MD, Kaiser WJ, Mocarski ES, Rothenberg ME, Hise AG, Dubyak GR, Ransohoff RM, Li X (2016). T cell-intrinsic ASC critically promotes T(H)17-mediated experimental autoimmune encephalomyelitis. Nat Immunol.

[49] Prada-Arismendy J, Arroyave JC, Rothlisberger S (2017). Molecular biomarkers in acute myeloid leukemia. Blood Rev.

[50] Poh AR, O'Donoghue RJ, Ernst M (2015). Hematopoietic cell kinase (HCK) as a therapeutic target in immune and cancer cells. Oncotarget.

[51] Dos Santos C, Demur C, Bardet V, Prade-Houdellier N, Payrastre B, Récher C (2008). A critical role for LYN in acute myeloid leukemia. Blood.

[52] Greenbaum D, Colangelo C, Williams K, Gerstein M (2003). Comparing protein abundance and mRNA expression levels on a genomic scale. Genome Biol.

[53] Vogel C, Marcotte EM (2012). Insights into the regulation of protein abundance from proteomic and transcriptomic analyses. Nat Rev Genet.

[54] Liu Y, Beyer A, Aebersold R (2016). On the dependency of cellular protein levels on mRNA abundance. Cell.

[55] Prochnicki T, Latz E (2017). Inflammasomes on the crossroads of innate immune recognition and metabolic control. Cell Metab.

[56] Saito Y, Yuki H, Kuratani M, Hashizume Y, Takagi S, Honma T, Tanaka A, Shirouzu M, Mikuni J, Handa N, Ogahara I, Sone A, Najima Y, Tomabechi Y, Wakiyama M, Uchida N, Tomizawa-Murasawa M, Kaneko A, Tanaka S, Suzuki N, Kajita H, Aoki Y, Ohara O, Shultz LD, Fukami T, Goto T, Taniguchi S, Yokoyama S, Ishikawa F (2013). A pyrrolo-pyrimidine derivative targets human primary AML stem cells in vivo. Sci Transl Med.

[57] Pene-Dumitrescu T, Peterson LF, Donato NJ, Smithgall TE (2008). An inhibitor-resistant mutant of HCK protects CML cells against the antiproliferative and apoptotic effects of the broad-spectrum c-SRC family kinase inhibitor A-419259. Oncogene.

[58] Naganna N, Opoku-Temeng C, Choi EY, Larocque E, Chang ET, Carter-Cooper BA, Wang M, Torregrosa-Allen SE, Elzey BD, Lapidus RG, Sintim HO (2019). Amino alkynylisoquinoline and alkynylnaphthyridine compounds potently inhibit acute myeloid leukemia proliferation in mice. EBioMedicine.

[59] Hu Y, Liu Y, Pelletier S, Buchdunger E, Warmuth M, Fabbro D, Hallek M, Van Etten RA, Li S (2004). Requirement of c-SRC kinases LYN, HCK and FGR for BCR-ABL1-induced B-lymphoblastic leukemia but not chronic myeloid leukemia. Nat Genet.

[60] Hobbs S, Jitrapakdee S, Wallace JC (1998). Development of a bicistronic vector driven by the human polypeptide chain elongation factor 1α promoter for creation of stable mammalian cell lines that express very high levels of recombinant proteins. Biochem Biophys Res Commun.

[61] Brakke MK (1951). Density Gradient Centrifugation: A New Separation Technique1. JACS.

[62] Schindelin J, Arganda-Carreras I, Frise E, Kaynig V, Longair M, Pietzsch T, Preibisch S, Rueden C, Saalfeld S, Schmid B, Tinevez JY, White DJ, Hartenstein V, Eliceiri K, Tomancak P, Cardona A (2012). Fiji: an open-source platform for biological-image analysis. Nat Methods.

[63] Cerami E, Gao J, Dogrusoz U, Gross BE, Sumer SO, Aksoy BA, Jacobsen A, Byrne CJ, Heuer ML, Larsson E, Antipin Y, Reva B, Goldberg AP, Sander C, Schultz N (2012). The cBio cancer genomics portal: an open platform for exploring multidimensional cancer genomics data. Cancer Discov.

