# Supplementary material for: Nucleotide binding domain and leucine-rich repeat pyrin domain-containing protein 12: characterization of its binding to hematopoietic cell kinase
Source: Int J Biol Sci. 2020 Mar 5;16(9):1507–25. doi: 10.7150/ijbs.41798 (PMC7097926; doi:10.7150/ijbs.41798)
Supplement: Supplementary file 1 — Supplementary figure and table. [file ijbsv16p1507s1.pdf]

Supplementary materials

Table S1: Complete yeast two hybrid results

Name	Frequency
Deleted in azoospermia associated protein 2 (DAZAP2)	10
Filamin A, alpha (FLNA)	9
Hematopoietic cell kinase proto-oncogene, Src family tyrosine kinase (HCK)	9
Chaperone DnaJ (a.k.a., Hsp40) homolog, subfamily B, member 1 (DNAJB1)	9
Metallothionein 2A (MT2A)	7
Cluster of differentiation 14 molecule (CD14)	6
DEAD (Asp-Glu-Ala-Asp) box helicase 5 (DDX5)	6
Talin 1 (TLN1)	5
Calcium modulating ligand (CAMLG)	4
Squamous cell carcinoma antigen recognized by T cells (SART1)	4
Ras-related nuclear protein binding protein 9 (RANBP9)	3
Tumor necrosis factor receptor associated factor 3 interacting protein 3 (TRAF3IP3)	3
Thyroid hormone receptor interactor 4 (TRIP4)	3
Ribosomal protein S20 (RPS20)	2
Coronin, actin binding protein, 1A (CORO1A)	2
Glutamate-ammonia ligase (GLUL)	2
Actin, beta (ACTB)	2
WW domain containing adaptor with coiled-coil (WAC)	2
Drosha, ribonuclease type III (DROSHA)	2
DnaJ (Hsp40) homolog, subfamily A, member 3 (DNAJA3)	1
Protein tyrosine phosphatase, receptor type, E (PTPRE)	1
Ubiquilin 1 (UBQLN1)	1
Adenylate cyclase-associated protein 1 (CAP1)	1
A kinase (protein kinase A (PRKA)) interacting protein 1 (AKIP1)	1
Ubiquilin 2 (UBQLN2)	1
Baculoviral inhibitor of apoptosis (IAP) repeat containing 3 (BIRC3)	1
Trinucleotide repeat containing 6C (TNRC6C)	1
Alkaline phosphatase, liver/bone/kidney (ALPL)	1
Pre-mRNA processing factor 6 (PRPF6)	1
Ubiquitin-like (UBX) domain protein 2B (UBXN2B)	1
B-cell lymphoma (BCL) 2-associated athanogene 6 (BAG6)	1
Secreted and transmembrane 1 (SECTM1)	1
Spermidine/spermine N1-acetyltransferase family member 2 (SAT2)	1
Zinc finger protein 426 (ZNF426)	1
Major histocompatibility complex, class I, B (HLA-B)	1
Fibrillin 2 (FBN2)	1
SH3-domain binding protein 2 (SH3BP2)	1
Myosin IF (MYO1F)	1
Ferritin, heavy polypeptide 1 (FTH1)	1
Leucine rich repeat containing 42 (LRRC42)	1
Fermitin family member 3 (FERMT3)	1
Lectin, galactoside-binding, soluble, 9B (LGALS9B)	1
Superoxide dismutase 2, mitochondrial (SOD2)	1
Carbohydrate (N-acetylgalactosamine 4-sulfate 6-O) sulfotransferase 15 (CHST15)	1
Hematopoietic cell-specific Lyn substrate (HCLS) 1 associated protein X-1 (HAX1)	1
Major histocompatibility complex, class I, C (HLA-C)	1
Splicing factor, arginine/serine-rich 17A (SFRS17A) (a kinase anchoring protein 17A)	1
Interleukin 8 (IL-8)	1

Supplementary materials Figure S1

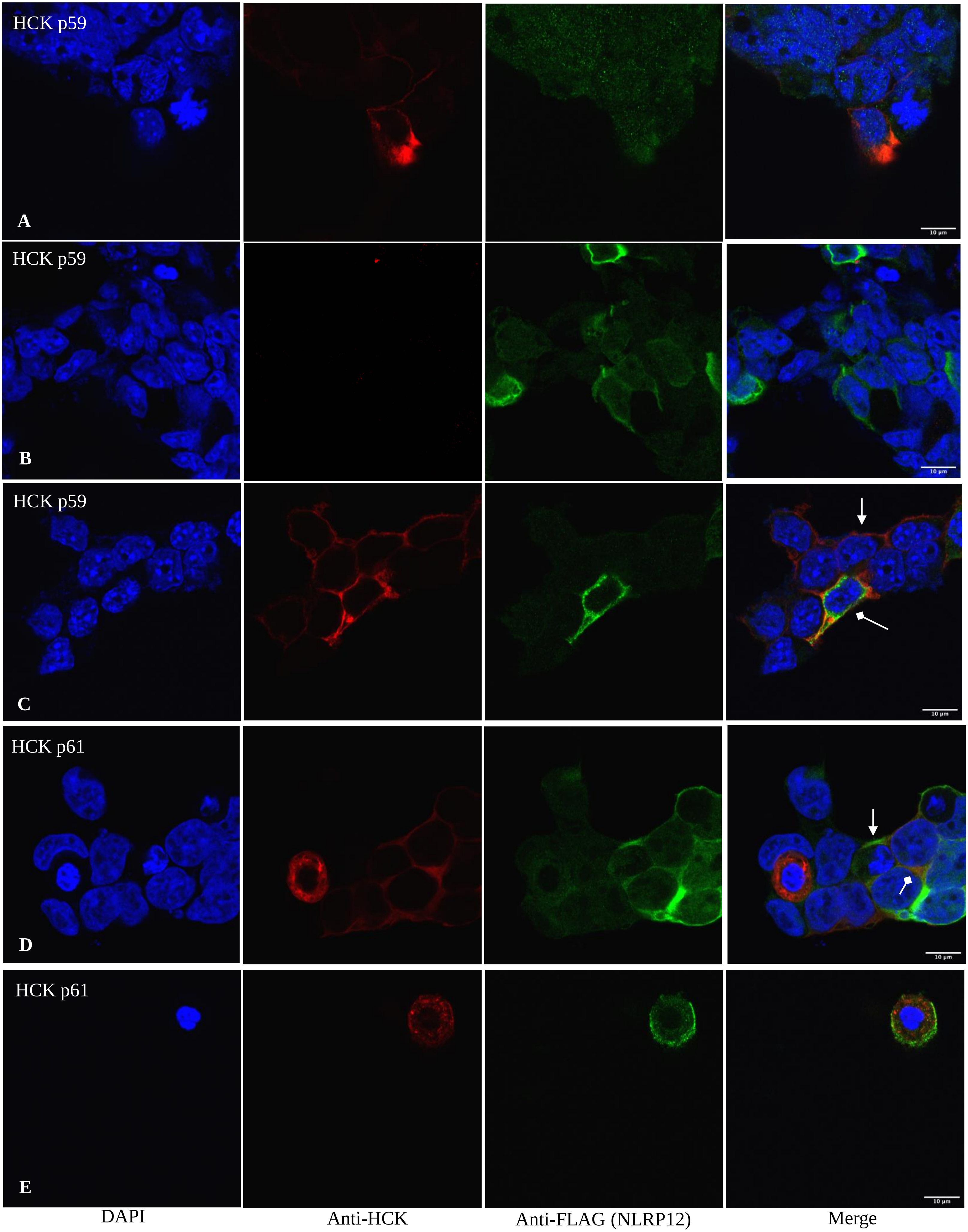

Supplementary materials Figure S1 legend

Figure S1: Immunofluorescent image of epitope-tagged NLRP12-3FLAG and HCK co-transfected into 293T cells, and fluorescently immunolabeled with anti-HCK and anti-FLAG (NLRP12) antibodies, and counterstained with DAPI. (A) NLRP12 only transfected into 293T cells. (B) HCK p61 only transfected into 293T cells. (C) - (E) shows that NLRP12 and HCK were co-transfected into and co-expressed in 293T cells. Panels (A) and (B) are negative controls for panel (E). Panels (C) and (D) indicate that some cells apparently only express Hck, while some cells only express NLRP12 (indicated by triangle arrow). And some cells co-express NLRP12 and HCK (indicated by square arrow). Panel (E) indicates that some cells express both NLRP12 and HCK, but they are not extensively co-localized.
